# Plasma lipid levels and risk of primary open angle glaucoma: a genetic study using Mendelian randomization

**DOI:** 10.1186/s12886-020-01661-0

**Published:** 2020-10-02

**Authors:** Mengqiao Xu, Shengguo Li, Jundong Zhu, Dawei Luo, Weitao Song, Minwen Zhou

**Affiliations:** 1 Department of Ophthalmology, Shanghai First People’s Hospital, School of Medicine, Shanghai JiaoTong University, 100 Haining Road, Shanghai, 200080 China; 2 Shanghai Key Laboratory of Fundus Disease, Shanghai, China; 3 Department of Ophthalmology, The Second Xiangya Hospital of Central South University, Changsha, China; 4 Department of Ophthalmology, the First People’s Hospital of Chenzhou, Southern Medical University, Chenzhou, China; 5 Eye Center of Xiangya Hospital, Central South University; Hunan Key Laboratory of Ophthalmology, Changsha, China

**Keywords:** Primary open angle glaucoma, Plasma lipid, Mendelian randomization

## Abstract

**Background:**

The causal effects of plasma lipid concentrations and the risk of primary open angle glaucoma (POAG) are still unclear. Thus, the purpose of this study was to identify, applying a two-sample Mendelian randomization (MR) analysis, whether plasma lipid concentrations are causally associated with the risk of POAG.

**Methods:**

Two-sample MR analysis of data from a genome-wide association study (GWAS) was performed to investigate the causal role of plasma lipid levels and POAG. A total of 185 independent single-nucleotide polymorphisms (SNPs) associated with plasma lipid levels were selected as instrumental variables (IVs). The SNPs were obtained from a meta-analysis of GWAS based on 188,577 European-ancestry individuals for MR analyses. Association with POAG for the SNPs was obtained from a GWAS conducted among the United Kingdom (UK) Biobank study participants with a total of 463,010 European-ancestry individuals. Four MR methods (inverse variance weighted [IVW], weighted mode, weighted median, and MR-Egger regression) were applied to obtain the overall causal estimate for multiple, instrumental SNPs.

**Results:**

Using the IVW analysis method, no evidence was found to support a causal association between plasma LDL-C level and POAG risk (β = − 0.00026; 95% CI = -0.00062, 0.00011; *P* = 0.165) with no significant heterogeneity among SNPs. The overall causal estimate between plasma LDL-C level and POAG was consistent using the other three MR methods. Using the four MR methods, no evidence of an association between plasma HDL-C (β = 0.00023; 95% CI = -0.00015, 0.00061; *P* = 0.238; IVW method) or TG levels (β = − 0.00028; 95% CI = -0.00071, 0.00015; *P* = 0.206; IVW method) and POAG risk was found. Sensitivity analyses did not reveal any sign of directional pleiotropy.

**Conclusions:**

The present study did not find any evidence for a causal association between plasma lipid levels and POAG risk. Further research is needed to elucidate the potential biological mechanisms to provide a reasonable interpretation for these results.

## Background

Primary open angle glaucoma (POAG), a frequent type of glaucoma worldwide, is a complicated disorder characterized by the progressive permanent degeneration of retinal ganglion cells (RGCs) and distinctive visual field loss [1, 2]. The worldwide prevalence has been estimated to 37.7 million people in 2020 and 74% will have POAG [3]. With the increasing burden of POAG, it is momentous to make clear the potentially risk factors for better prevention. To date, in addition to elevated intraocular pressure (IOP) [4, 5], other risk factors, such as age [6, 7], diabetes [8], hyperlipidemia [9], family history [10], and ethnic background [11], have been established in connection with the risk of POAG. However, such relationships can be influenced by confounding from unknown factors. Thus, it is hard to clarify the causal risk factors.

Several epidemiological studies have reported plasma lipid concentrations and hyperlipidemia have a significant association with the risk of POAG [12–14]. A meta-analysis aggregating data from 18 epidemiological studies identified that hyperlipidemia caused a 37% increase in the risk of patients developing POAG [9]. The quantitative analysis in this meta-analysis also showed that an increase of 10 mg/dl in the plasma triglyceride concentration, plasma total cholesterol concentration, or plasma low-density lipoprotein cholesterol (LDL-C) concentration would increase the IOP by 0.016 mmHg, 0.032 mmHg, or 0.050 mmHg, respectively [9]. Nevertheless, these relationships may be biased because of the inherent limitations of epidemiological studies such as reverse causation, confounding, and measurement error [15]. Evidence that clarifies the association between plasma lipid and POAG is needed urgently for better prevention.

Mendelian randomization (MR) is an alternative method for potential causal inference that treats genetic variations as a natural experiment in which individuals are essentially assigned to higher vs. lower mean levels of a nongenetic exposure during their lifetime [16]. A single-nucleotide polymorphism (SNP) is a DNA sequence variation occurring when a single nucleotide adenine (A), thymine (T), cytosine (C), or guanine (G) in the genome (or other shared sequence) differs among members of a species or paired chromosomes in an individual. Due to Mendel’s laws of inheritance and the fixed nature of germline genotypes, the alleles an individual receives at this SNP are expected to be random, with respect to potential confounders and causally upstream of the exposure. Thus, MR, an analytical approach that uses genetic variants (SNPs) as instruments, has been widely used to estimate the potential causality between a risk factor and outcome [16]. In recent years, a growing number of genome-wide association studies (GWASs) have been done and the data from GWASs can be used to perform two-sample MR analysis to estimate the causal effects where gene exposure and gene outcome are measures in different samples. For example, Fan et al. [17]. used the two-sample MR method to determine that high levels of plasma HDL-C are causally associated with an increased risk for advanced age-related macular degeneration (AMD) in European and Asian populations. To date, the two-sample MR analysis has not been applied to explore the causal effects of plasma lipid concentrations and the risk of POAG. Thus, the purpose of our study was to identify, applying a two-sample MR analysis, whether plasma lipid concentrations are causally associated with the risk of POAG.

## Methods

### Study design

**Fig. 1:**
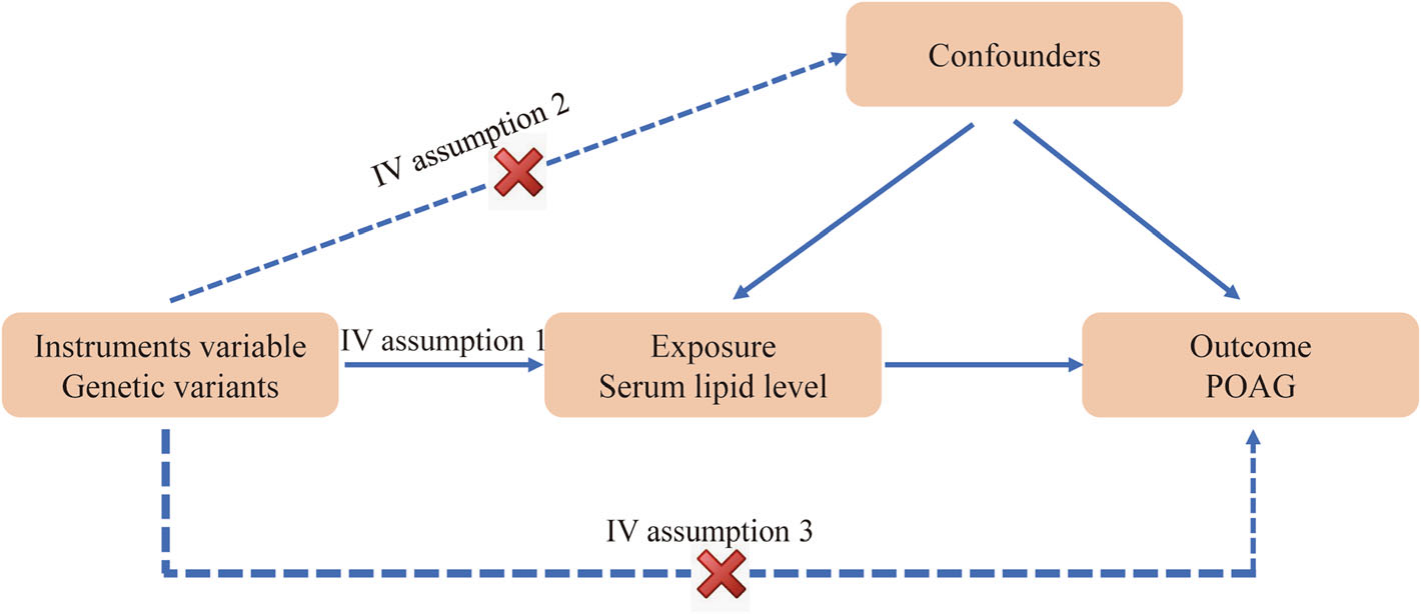
The explanation of Mendelian randomization (MR) analysis by a directed acyclic graph. The accuracy of estimating causality using MR analyses is based on the following three assumptions: (1) The instrumental variable (IV) associates robustly with the exposure (IV assumption 1). This assumption can be satisfied in that SNPs are selected using GWAS levels (*P* < 5 × 10^− 8^), which suggests that potential bias from weak IV should not be substantial. (2) The IV is independent of the combined influence of all confounders (IV assumption 2). For the same population and reference, we assess the correlation of linkage disequilibrium between SNPs associated robustly with exposure. If the correlation coefficient is higher, the corresponding selected SNPs will be discarded. (3) The IV is independent of the outcome given the exposure and confounders (IV assumption 3). Horizontal pleiotropy (that IVs influence the outcome through alternative pathways other than the exposure) could violate this assumption. This assumption can be checked by using MR-Egger regression

These two-sample MR analyses were conducted based on de-identified summary-concentrations data that have been made publicly available; ethical approval was obtained in all original studies. MR analyses assessed the casual effect of an exposure on an outcome by using SNPs as instrumental variables (IVs), in the presence of unmeasured confounding, given that the genotypes are conditionally independent of the disease status [17–19]. The following assumptions are made for MR inference: first, the genetic variant has a robust causal relationship with the exposure; second, the genetic variant affects the outcome only through its effect on the exposure; third, the genetic variant and the outcome do not have common causes (Fig. 1) [20]. In the present study, two-sample MR analyses with four stages were employed. First, we evaluated whether SNPs were independently associated with exposure. Second, we explored whether each SNP was associated with the outcomes. Third, we combined these findings to assess the unconfounded causative relationship between exposure and outcomes. Fourth, we performed sensitivity analyses to confirm the robustness of our findings.

### Data sources and instruments

Two-sample MR analyses were performed using GWAS summary data. These two GWAS datasets were required to have a similar genetic ancestry. Three plasma lipid fractions— LDL-C, high-density lipoprotein cholesterol (HDL-C), and triglycerides (TG)—were included in this study as the exposure variables. For the exposure dataset, a publicly available summary statistics data, based on 188,577 European-ancestry individuals, were identified through a meta-analysis of GWAS from the Global Lipids Genetics Consortium (GLGC) (Sample 1) [21, 22]. For the outcome dataset, we extracted the summary statistic datasets from a recent GWAS of POAG conducted among the United Kingdom (UK) Biobank study participants with a total of 463,010 European-ancestry individuals (Sample 2) and accessed through the MR-Base database (http://www.mrbase.org/). The MR-Base is home to a huge collection of summary data from many GWASs [23]. For the two-sample MR, it was important to certify that the IVs for the exposure were robustly independent. Therefore, we examined the clumping test to assess the linkage disequilibrium (LD) (*r*^*2*^ threshold of 0.001). In addition, if a particular SNP is not obtained in the outcome dataset, then we will use SNPs that are LD ‘proxies’ instead; these lookups are automatically provided by MR-Base.

In the GWASs of GLGC, a total of 185 independent SNPs robustly associated with plasma lipid concentrations reached a threshold of genome-wide significance (*P* < 5.0 × 10^− 8^) and were selected as IVs for MR analysis (Supplementary Table 1). These 185 SNPs accounted for 6.9% of the variance in LDL-C, 6.4% of the variance in HDL-C, and 5.2% of variance in TG. Of these selected SNPs, 82 were associated with LDL-C, 96 were associated with HDL-C, and 60 were associated with TG. From amongst these 82 SNPs associated with LDL-C, 26 (rs1998013, rs1010167, rs903319, rs646776, rs515135, rs3817588, rs4148218, rs2030746, rs2247056, rs2297374, rs4722551, rs217386, rs10102164, rs2980885, rs8176720, rs174532, rs11220462, rs6489818, rs1186380, rs2288002, rs4791641, rs688, rs10401969, rs6859, rs7264396, rs6016381) were excluded as potential instrumental variables for having a pairwise LD coefficient of determination (*r*^*2*^) > 0.001 with another variant in the set. From amongst these 96 SNPs associated with HDL-C, 24 (rs6680658, rs355838, rs13326165, rs442177, rs6450176, rs17286602, rs4332136, rs894210, rs4871137, rs2980885, rs2472509, rs17788930, rs11246602, rs12226802, rs12801636, rs653178, rs10773105, rs2412710, rs261342, rs2652834, rs5880, rs11660468, rs1800961) were excluded as potential instrumental variables for having a pairwise LD *r*^*2*^ > 0.001 with another variant in the set. From amongst these 60 SNPs associated with TG, 14 (rs10493326, rs3817588, rs10029254, rs799160, rs4921914, rs894210, rs2980885, rs603446, rs2412710, rs261342, rs2652834, rs9930333, rs5880, rs1688030) were excluded as potential instrumental variables for having a pairwise LD *r*^*2*^ > 0.001 with another variant in the set.

### Statistical analyses

The primary analyses of causal relationship of IVs for plasma lipid concentrations with POAG were performed using the standard inverse variance weighted (IVW) method. In addition to the IVW method, if other MR models (weighted median estimator, MR-Egger regression, and weighted mode-based estimator), which make different assumptions regarding instrument validity, were to also produce similar estimates of the causal effect, then we can be more confident in the robustness of our findings. Because the IVW method would yield biased estimates in the presence of horizontal pleiotropy, several sensitivity analyses were conducted to assess validity of MR findings. First, different MR analyses were used to verify with orthogonal MR methods (IVW, MR-Egger, weighted mode, and median-weighted MR) [24–26]. Second, MR-Egger intercept was used for detection of and adjustment for directional pleiotropy. A third sensitivity analysis was performed by omitting a single SNP each time (leave-one-out analysis). The heterogeneities among SNPs were assessed using Cochran’s Q test and funnel plots. All the analyses were performed using the MR-Base platform (http://app.mrbase.org/) [23]. Plots such as forest plot, scatter plots, and funnel plots were also generated using this platform. Plots such as forest plot and scatter plots were also generated using this platform. All statistical analyses used a *P* < 0.05 threshold to indicate statistical significance.

## Results

### The selection and validation of instrumental variables

There were 82, 96, and 60 index SNPs, respectively, associated with LDL-C, HDL-C, and TG separately (Supplementary Table 1). However, 26 of 82, 23 of 96, and 14 of 60 SNPs that were related to LDL-C, HDL-C, and TG separately were removed because of a pairwise LD *r*^*2*^ > 0.001. Thus, the remaining SNPs associated independently with exposure were chosen as IVs for further MR analyses (Supplementary Tables 2, 3, 4).

### Causal effect from LDL-C to POAG

**Fig. 2:**
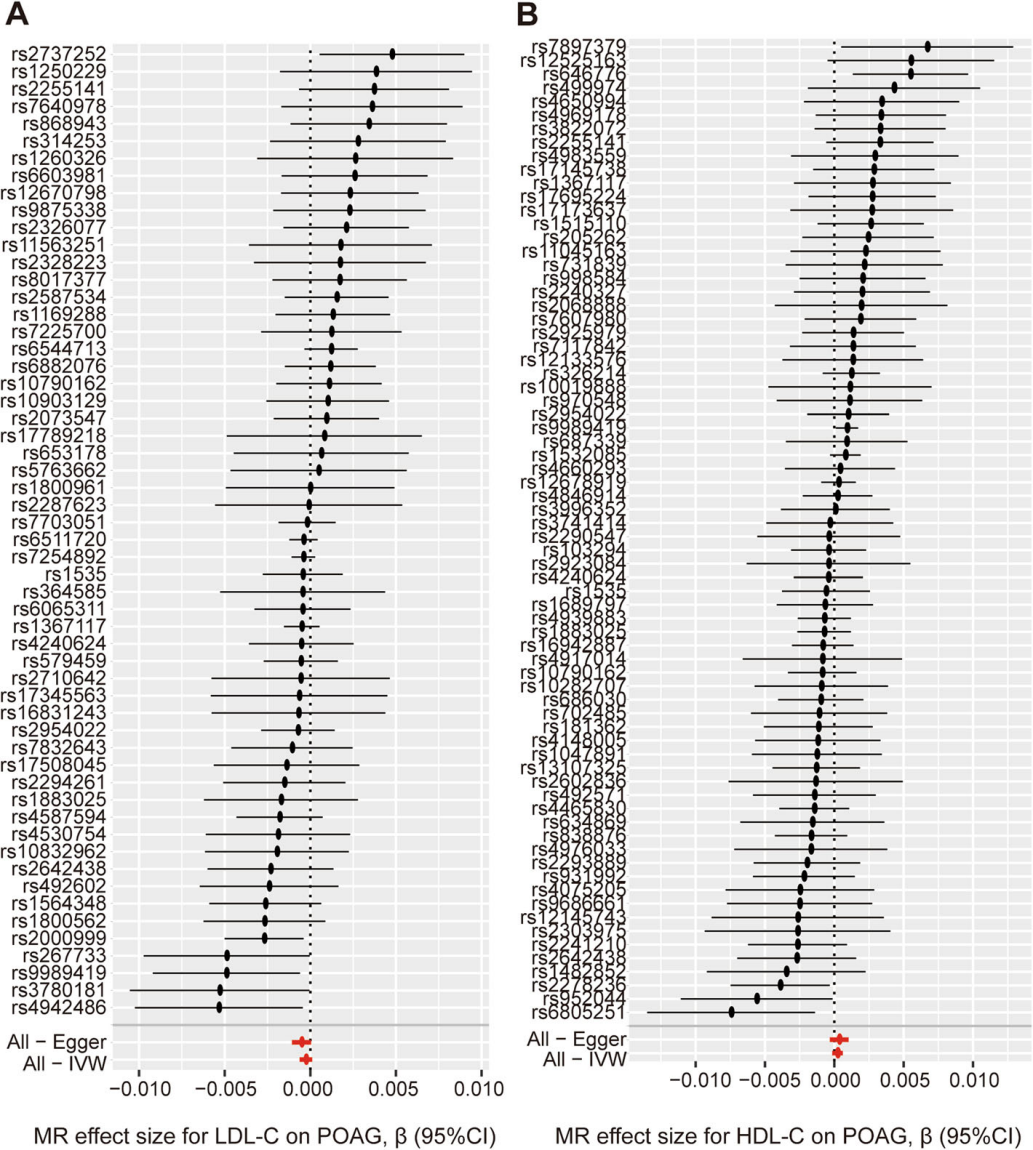
Forrest plot of the causal effects of plasma LDL-C level (**a**) or plasma HDL-C level (**b**) associated SNPs on POAG

**Fig. 3:**
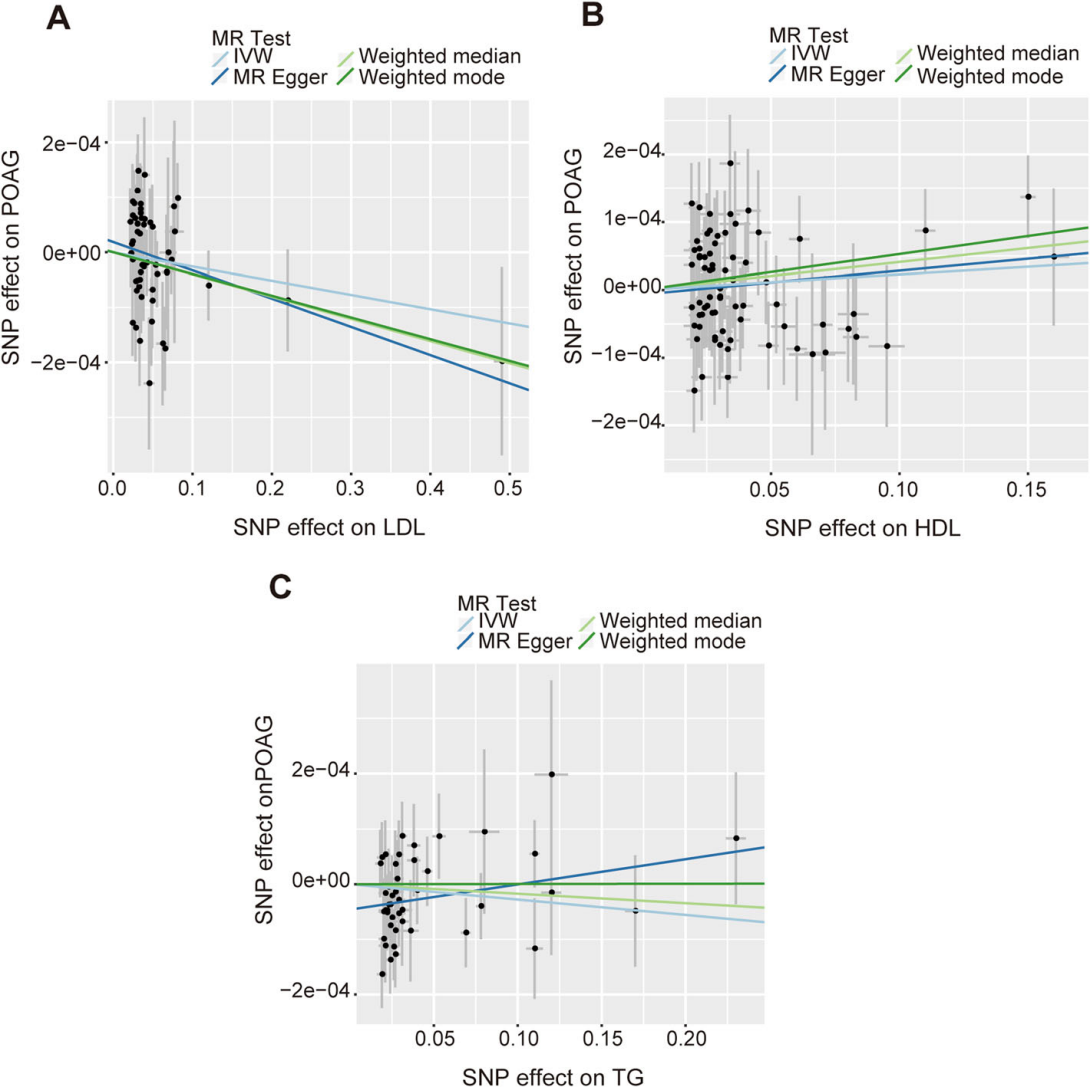
Scatter plots of the genetic associations of plasma LDL-C level (**a**), or plasma HDL-C level (**b**) or plasma TG level (**c**) associated SNPs against the genetic associations of POAG. The slopes of each line represent the causal association for each method

**Fig. 4:**
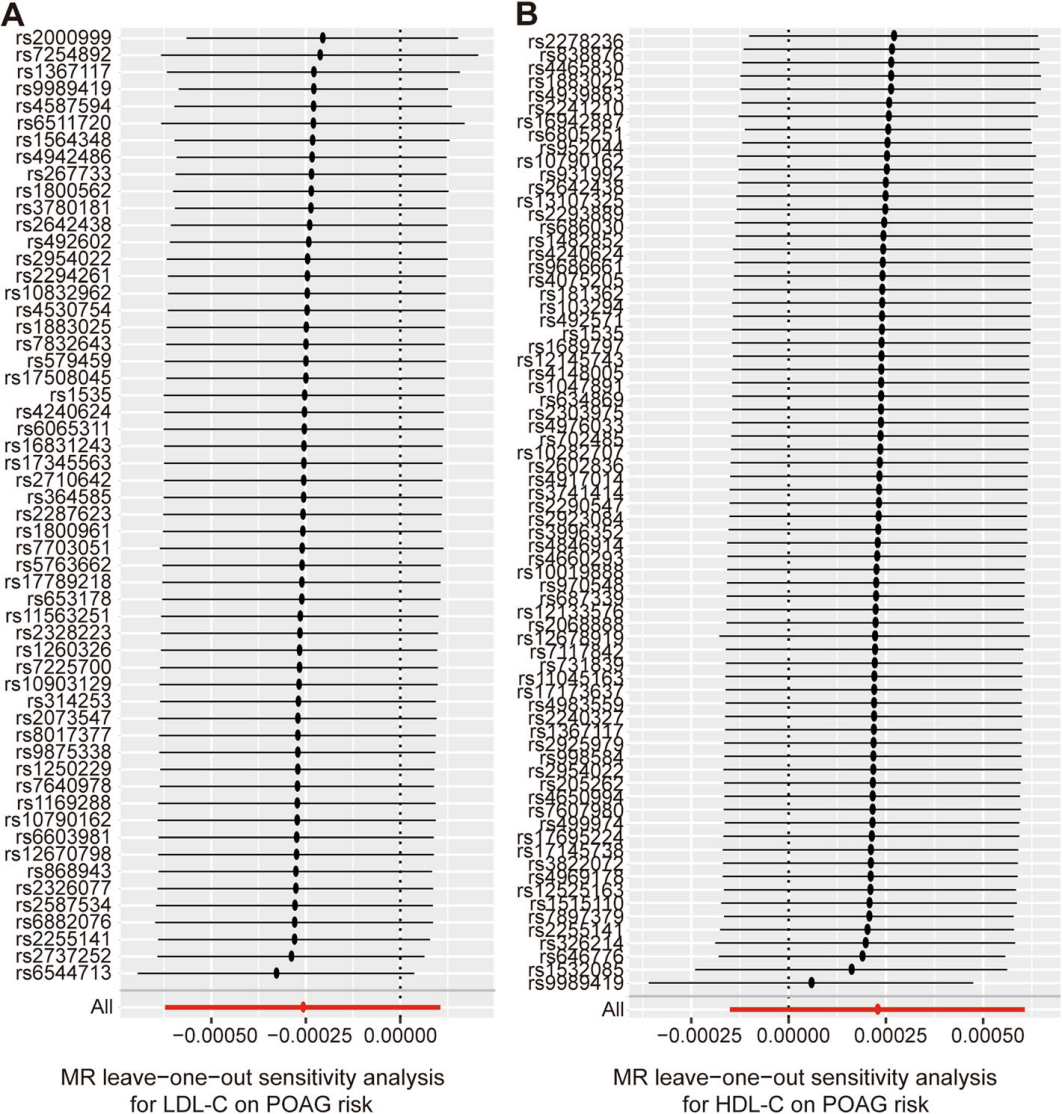
Leave-one-out permutation analysis plots for plasma LDL-C level (**a**) or plasma HDL-C level (**b**) on POAG risk obtained by leaving out the SNP indicated and repeating the standard inverse-variance weighted method with the rest of the 55 and 71 SNP IVs used respectively

The results of the MR analysis are presented in Table 1. In the MR analysis of LDL-C level and POAG, the overall causal estimate from the IVW method suggested a null effect on risk of POAG per SD change in each LDL-C (β = − 0.00026; 95% CI = -0.00062, 0.00011; *P* = 0.165) (Figs. 2a, 3a). The Cochran Q test showed that no significant heterogeneity was detected among SNPs (Q = 66.23; *P* = 0.143). As expected, the overall causal estimate between plasma LDL-C level and POAG was consistent in sensitivity analyses using MR-Egger (β = − 0.00051; 95% CI = -0.00107, 0.00005; *P* = 0.078), weighted median (β = − 0.00040; 95% CI = -0.00088, 0.00008; *P* = 0.099) and weighted mode method (β = − 0.00039; 95% CI = -0.00087, 0.00008; *P* = 0.111) (Table 1). To assess the influence of an individual SNP on the pooled results, one SNP was omitted at a time. The corresponding estimates of plasma LDL-C level on POAG were not significantly influenced by any single SNP, reflecting the high stability and reliability of the results (Fig. 4a). In the MR-Egger intercept analysis, the result suggested an absence of significant horizontal pleiotropy (intercept = 1.9 × 10^− 5^; SE = 1.6 × 10^− 5^; *P* = 0.246) (Table 2). These results were consistent with the hypothesis that genetic pleiotropy was not driving the result.

**Table 1:** Mendelian randomization estimates of the associations between serum lipid level and risk of POAG

Exposure	Method	n. SNP	β	95%CI	***p***
LDL-C	IVW	56	−0.00026	− 0.00062, 0.00011	0.165
LDL-C	MR Egger	56	−0.00051	−0.00107, 0.00005	0.078
LDL-C	Weighted median	56	−0.00040	−0.00088, 0.00008	0.099
LDL-C	Weighted mode	56	−0.00039	−0.00087, 0.00008	0.111
HDL-C	IVW	72	0.00023	−0.00015, 0.00061	0.238
HDL-C	MR Egger	72	0.00035	−0.00032, 0.00102	0.310
HDL-C	Weighted median	72	0.00041	−0.00015, 0.00097	0.151
HDL-C	Weighted mode	72	0.00053	−0.00005, 0.00111	0.077
TG	IVW	46	−0.00028	−0.00071, 0.00015	0.206
TG	MR Egger	46	0.00046	−0.00018, 0.00110	0.170
TG	Weighted median	46	−0.00017	−0.00076, 0.00042	0.569
TG	Weighted mode	46	3.63E-06	−0.00055, 0.00056	0.990

*POAG* Primary open angle glaucoma, *SNP* Single nucleotide polymorphism, *LDL* Low density lipoprotein cholesterol, *IVW* Inverse variance weighted, *HDL* High density lipoprotein cholesterol, *TG* Triglycerides, *β* The effect of the effect allele, *CI* Confidence interval, *p P*-value from the GWAS

**Table 2:** MR-Egger pleiotropy test of the associations between serum and risk of POAG

Exposure	Intercept	se	***p***
LDL-C	1.9E-05	1.6E-05	0.246
HDL-C	−6.50E-06	1.50E-05	0.665
TG	−4.60E-05	1.60E-05	0.006

*MR* Mendelian randomization, *POAG* Primary open angle glaucoma, *LDL* Low density lipoprotein cholesterol, *HDL* High density lipoprotein cholesterol, *TG* Triglycerides, *se* Standard error, *p P*-value from the GWAS

### Causal effect from HDL-C to POAG

When we assessed the causal relationship between HDL-C level with POAG, no evidence of an association was detected using the IVW analysis method (β = 0.00023; 95% CI = -0.00015, 0.00061; *P* = 0.238) (Table 1) (Figs. 2b, 3b). Non-significant heterogeneity was detected across the instrument SNP effects (Q = 79.99; *P* = 0.218). MR-Egger, weighted median, and weighted mode analysis yielded similar patterns of effects (Table 1). In a leave-one-out sensitivity analysis, we found that no single SNP influenced significantly the overall effect of HDL-C on POAG (Fig. 4b).

The MR-Egger intercept test did not reveal any signs of horizontal pleiotropy for HDL-C level (intercept = − 6.5 × 10^− 6^; SE = 1.5 × 10^− 5^; *P* = 0.665) (Table 2).

### Causal effect from TG to POAG

**Fig. 5:**
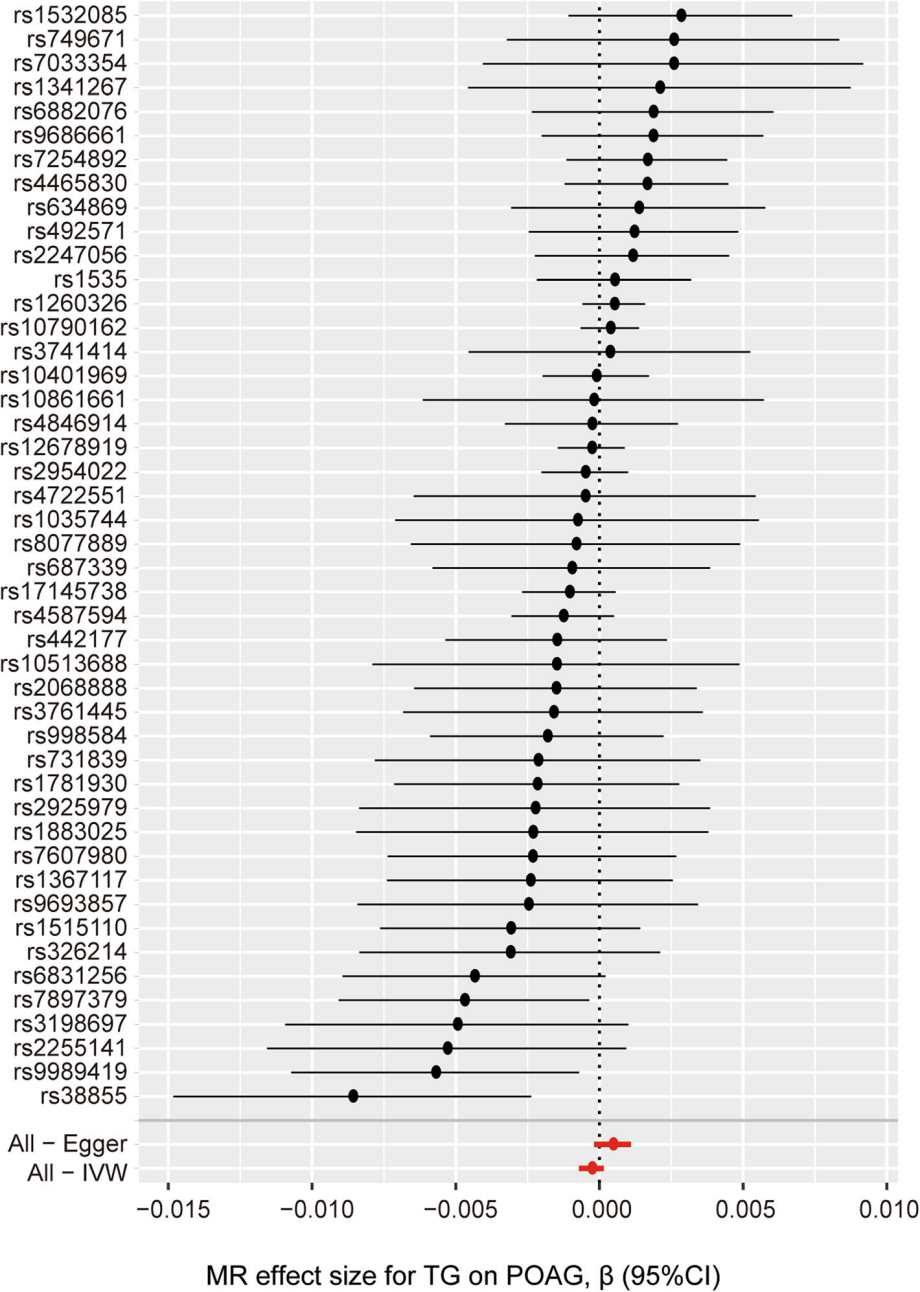
Forrest plot of the causal effects of plasma TG level associated SNPs on POAG

**Fig. 6:**
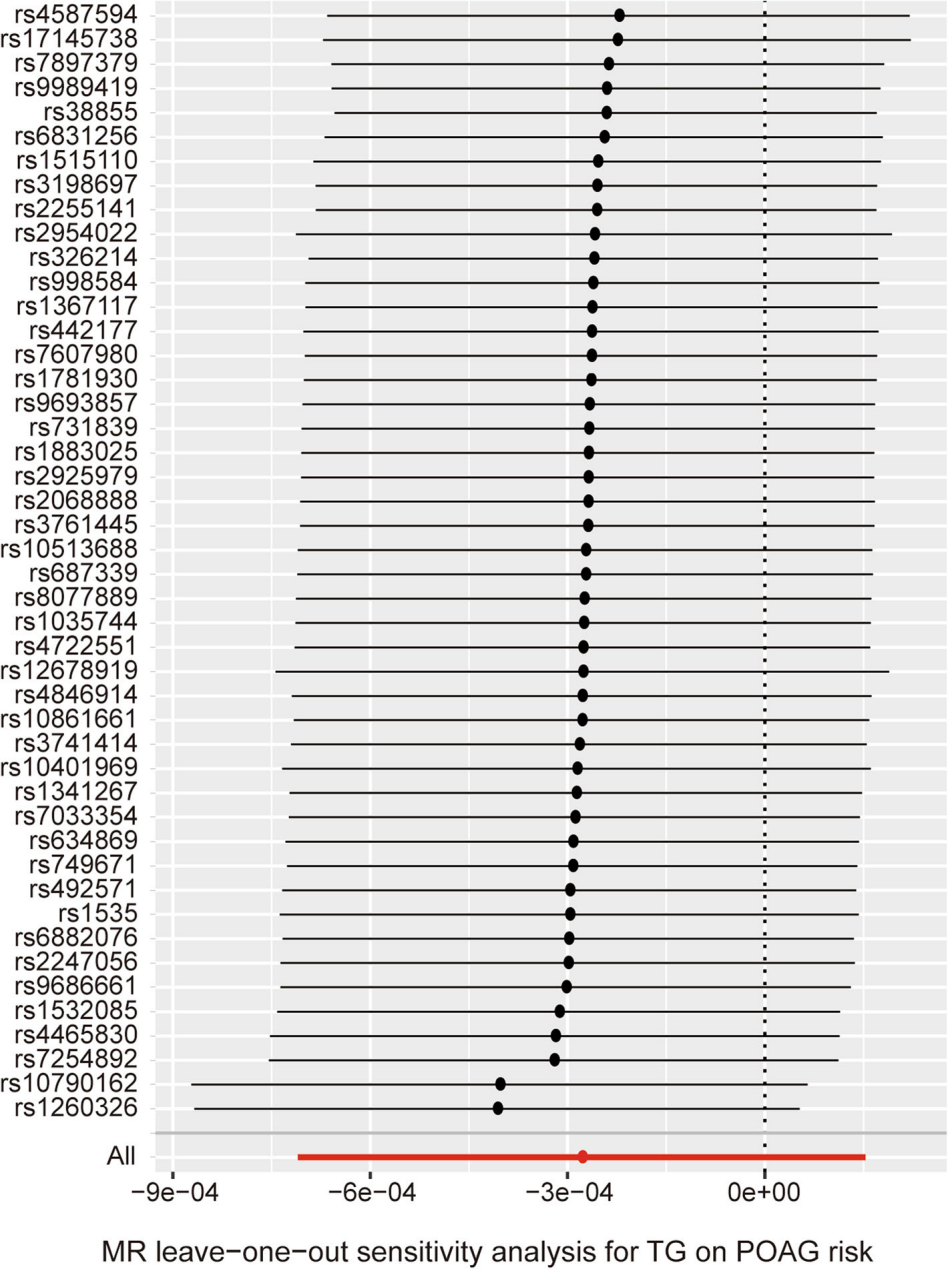
Leave-one-out permutation analysis plots for plasma TG levels on POAG risk obtained by leaving out the SNP indicated and repeating the standard inverse-variance weighted method with the rest of the 45 SNP IVs used respectively

The causal associations of genetically predicted TG level with POAG based on the IVW method presented a negative result (β = − 0.00028; 95% CI = -0.00071, 0.00015; *P* = 0.206) (Table 1) (Figs. 3c, 5). There was no significant heterogeneity across the instrument SNPs (Q = 49.57; *P* = 0.296). The negative relationships also remained when using the MR-Egger method (β = 0.00046; 95% CI = -0.00018, 0.00110; *P* = 0.170). In analyzing the association between the TG level and POAG using the median-weighted, mode-based estimate, the result presented remained negative (Table 1). The MR-Egger regression analysis also did not reveal any signs of directional pleiotropy (intercept = − 6.5 × 10^− 6^; SE = 1.5 × 10^− 5^; *P* = 0.665) (Table 2). Subsequently, a leave-one-out sensitivity analysis was also performed to assess the robustness of this findings and the result showed that no single SNP significantly influenced the overall effect of TG level on POAG (Fig. 6).

## Discussion

POAG is a common disorder, with a high societal burden [27]. Thus, identification of effective approaches for preventing the development of POAG is very necessary. Previous observational studies have shown contradictory results for relationships between plasma lipid levels and the risk of POAG [28–30]. A newly published meta-analysis performed by Wang et al. [9] found a significant association between plasma lipid levels and the risk of POAG (not including normal tension glaucoma). However, considering these observational studies are limited by potential residual confounding and reverse causality, we should interpret these findings with caution. Here, we performed a two-sample MR analysis with genetic instruments selected from large-scale GWAS to assess whether a causal association exists between plasma lipids levels and the risk of POAG on the basis of genetic data from European-ancestry populations. The findings suggested that plasma LDL-C, HDL-C, and TG levels were not found to be related with the risk of POAG; this was done using four different estimation methods after adjusting for genetic linkage (IVW, weighted median, weighted mode, and MR-Egger regression). To the best of our knowledge, this is the first study to clarify a causal association between plasma lipids levels and the risk of POAG using the MR method, which provides greater freedom from confounder bias than the risks reported in the observational epidemiological studies carried out to date [31].

Concerning the relationship between plasma lipids levels and POAG, Wang et al. [9] clarified that hyperlipidemia is significantly associated with an increased risk of glaucoma based on pooled responses across 18 epidemiological studies. Wang et al. speculated that the possible explanation of this association might be that excess blood lipid levels would increase the episcleral venous pressure and blood viscosity, resulting in a consequent decrease in outflow facility [9, 32]. Nevertheless, despite the reasonable biological rationale, the causality for the association between plasma lipid levels and the risk of POAG has remained unclear. Our findings are contrary to the previous meta-analysis results and are also contrary to a longitudinal cohort study with a large sample size of 2,182,315, which suggested that individuals with hyperlipidemia had a reduced risk of developing POAG compared to those with no hyperlipidemia [28]. They explained that the hyperlipidemia itself, or the medications used to treat hyperlipidemia, or both might decrease the incidence of POAG [28]. In our study, the association between plasma lipid levels and POAG risk were measured using MR analysis, which decreases bias by reason of confounding such as the medications used to treat hyperlipidemia. The positive or inverse association between the plasma lipid level and POAG risk proven by previous observational studies was likely affected by other confounders, such as hypertension and diabetes.

A key assumption for MR is that the effect of an SNP on outcomes is mediated through its influence on the exposure [33]. However, horizontal pleiotropy in MR studies has to be issued. Horizontal pleiotropy occurs always when the genetic variants influence the outcome through another pathway instead of the one under analysis [34]. If the horizontal pleiotropy is directional, all traits have analogous effects on the outcome and will lead to an exaggerated MR estimate. In addition, because we were unlikely to make clear the biological actions of all the included SNPs, it is impossible to exclude pleiotropic mechanisms fully without a detailed functional follow-up of these SNPs. Moreover, SNPs related to lipid levels are likely to be associated with other lipid fractions and unknown pathways. Thus, it is possible that horizontal pleiotropy may be present in our study. We conducted sensitivity analyses to address this. The MR–Egger intercept analysis showed no evidence for unbalanced horizontal pleiotropy, and similar results were observed in IVW and weighted-median analyses, suggesting that the likelihood of the presence of horizontal pleiotropy is small.

The first strength of our study is the MR design, which reduces the risk of bias from confounding factors and reverse causality. The second strength is that our study included a relatively large number of genetic variants as IVs, which had sufficient statistical power to detect causal effects. Third, different methods including IVW, weighted-median, MR-Egger, and weighted-mode method were used in this 2-sample MR analysis, the causal estimates of which were consistent, thus enhancing the robustness of the result. Nevertheless, several limitations in this study should be disclosed. First, although some methods such as MR-Egger regression analysis and leave-one-out sensitivity analysis were carried out to minimize the risk of bias from the potential use of invalid instrumental variables, such bias cannot be completely ruled out. Second, nearly all SNPs have only a limited effect on a given exposure, because SNPs for an exposure may explain only a small proportion of variance in that exposure [35]. Third, this MR analysis was based only on European-ancestry individuals. Because causality may depend on ethnicity, further MR studies are needed in other ethnic groups. Fourth, reasonable biological interpretations for the results of the MR analyses were not provided. Finally, it is important to note that selection of relevant SNPs as an IVs is important for successful MR study. MR analysis requires genetic variants to be related to, but not potential confounders, an exposure. Selected genetic variants must be independent and strongly associated with the exposure. Otherwise, weak instrument bias can happen in MR studies. Weak relationship between the risk SNPs and the exposure may prompt negative MR results, because of low statistical power to recognize a relationship. Thus, the results could not be considered as a definitive conclusion and should be generalized to the rest of the ethnic groups with caution.

## Conclusion

In summary, we carried out a 2-sample MR analysis using lipid-associated SNPs to investigate the influence of plasma lipid levels on POAG risk. The present study did not find any evidence for a causal association between plasma lipid levels and POAG risk. However, of note, patients with high level of lipids are more prone to other disease that might lead to the development of glaucoma. Because of the limitation of this study, experimental findings from biological mechanisms are expected to provide a reasonable interpretation for these results.

## Supplementary information

**Additional file 1: Table S1.** Genetic variants used as instrumental variables in the Mendelian randomization analysis estimating the causal effect of serum lipid levels on POAG.

**Additional file 2: Table S2.** Summary of selected instrumental variables for LDL-C.

**Additional file 3: Table S3.** Summary of selected instrumental variables for HDL-C.

**Additional file 4: Table S4.** Summary of selected instrumental variables for TG.

## Data Availability

The datasets used and/or analyzed during the current study are available from the corresponding author on reasonable request.

## Abbreviations

POAG: Primary open angle glaucoma
RGC: Retinal ganglion cell
IOP: Intraocular pressure
LDL-C: Low-density lipoprotein cholesterol
MR: Mendelian randomization
SNP: Single-nucleotide polymorphism
GWAS: Genome-wide association study
IV: Instrumental variable
HDL-C: High-density lipoprotein cholesterol
TG: Triglyceride
GLGC: Global Lipids Genetics Consortium
UK: United Kingdom
LD: Linkage disequilibrium
IVW: Inverse variance weighted
