# Supplementary material for: Plasma lipid levels and risk of primary open angle glaucoma: a genetic study using Mendelian randomization
Source: BMC Ophthalmol. 2020 Oct 2;20:390. doi: 10.1186/s12886-020-01661-0 (PMC7532556; doi:10.1186/s12886-020-01661-0)
Supplement: Supplementary file 1 — Additional file 1: Table S1. Genetic variants used as instrumental variables in the Mendelian randomization analysis estimating the causal effect of serum lipid levels on POAG. [file 12886_2020_1661_MOESM1_ESM.docx]

**Supplementary Table 1.** Genetic variants used as instrumental variables in the Mendelian randomization analysis estimating the causal effect of serum lipid levels on POAG.

| **SNP** | **CHR** | **BP** | **EA** | **NEA** | **EAF** | **β_HDL-C** | **se_HDL-C** | ***p*_HDL-C** | **β_LDL-C** | **se_LDL-C** | ***p*_LDL-C** | **β_TG** | **se_TG** | ***p*_TG** |
| --- | --- | --- | --- | --- | --- | --- | --- | --- | --- | --- | --- | --- | --- | --- |
| rs10903129 | 1 | 2.6E+07 | A | G | 0.463 | -0.001 | 0.003 | 7.90E-01 | -0.033 | 0.004 | 4.00E-19 | -0.008 | 0.003 | 1.70E-02 |
| rs4660293 | 1 | 4E+07 | A | G | 0.764 | 0.035 | 0.004 | 7.00E-19 | -0.011 | 0.004 | 1.40E-02 | -0.020 | 0.004 | 1.00E-07 |
| rs1998013 | 1 | 5.6E+07 | T | C | 0.008 | 0.035 | 0.020 | 7.70E-02 | -0.380 | 0.022 | 4.00E-67 | 0.009 | 0.020 | 6.60E-01 |
| rs10493326 | 1 | 6.3E+07 | A | G | 0.247 | -0.001 | 0.004 | 7.40E-01 | 0.021 | 0.004 | 6.00E-07 | 0.031 | 0.004 | 1.00E-15 |
| rs4587594 | 1 | 6.3E+07 | A | G | 0.310 | -0.015 | 0.004 | 5.00E-05 | -0.049 | 0.004 | 3.00E-37 | -0.069 | 0.003 | 3.00E-87 |
| rs6603981 | 1 | 9.3E+07 | T | C | 0.806 | 0.004 | 0.004 | 3.50E-01 | 0.034 | 0.004 | 2.00E-14 | 0.007 | 0.004 | 7.60E-02 |
| rs12133576 | 1 | 9.4E+07 | A | G | 0.355 | 0.024 | 0.003 | 6.00E-12 | 0.010 | 0.004 | 6.00E-03 | -0.009 | 0.003 | 8.80E-03 |
| rs646776 | 1 | 1.1E+08 | T | C | 0.788 | -0.034 | 0.004 | 9.00E-17 | 0.160 | 0.004 | 1.00E-292 | 0.003 | 0.004 | 3.90E-01 |
| rs1010167 | 1 | 1.1E+08 | C | G | 0.584 | 0.004 | 0.004 | 2.30E-01 | -0.025 | 0.004 | 3.00E-10 | -0.002 | 0.004 | 6.60E-01 |
| rs267733 | 1 | 1.5E+08 | A | G | 0.863 | -0.016 | 0.005 | 1.20E-03 | 0.033 | 0.005 | 5.00E-10 | 0.003 | 0.005 | 6.10E-01 |
| rs12145743 | 1 | 1.5E+08 | T | G | 0.669 | -0.020 | 0.004 | 2.00E-08 | 0.004 | 0.004 | 2.90E-01 | 0.012 | 0.004 | 6.00E-04 |
| rs4650994 | 1 | 1.8E+08 | A | G | 0.483 | -0.021 | 0.003 | 6.00E-10 | 0.003 | 0.004 | 4.50E-01 | 0.002 | 0.003 | 4.70E-01 |
| rs1689797 | 1 | 1.8E+08 | A | C | 0.302 | -0.036 | 0.004 | 2.00E-23 | 0.014 | 0.004 | 2.20E-04 | 0.011 | 0.004 | 2.50E-03 |
| rs2642438 | 1 | 2.2E+08 | A | G | 0.255 | -0.030 | 0.004 | 5.00E-15 | -0.035 | 0.004 | 4.00E-17 | 0.017 | 0.004 | 5.00E-06 |
| rs903319 | 1 | 2.2E+08 | T | C | 0.723 | -0.010 | 0.004 | 6.70E-03 | -0.027 | 0.004 | 8.00E-11 | 0.005 | 0.004 | 1.50E-01 |
| rs4846914 | 1 | 2.3E+08 | A | G | 0.584 | 0.048 | 0.003 | 6.00E-44 | -0.004 | 0.004 | 2.50E-01 | -0.040 | 0.003 | 7.00E-33 |
| rs6680658 | 1 | 2.3E+08 | A | G | 0.231 | 0.023 | 0.004 | 2.00E-08 | -0.006 | 0.004 | 2.20E-01 | -0.017 | 0.004 | 4.00E-05 |
| rs2587534 | 1 | 2.3E+08 | A | G | 0.528 | 0.009 | 0.003 | 6.50E-03 | 0.039 | 0.004 | 3.00E-26 | 0.004 | 0.003 | 2.20E-01 |
| rs1367117 | 2 | 2.1E+07 | A | G | 0.288 | -0.022 | 0.004 | 2.00E-09 | 0.120 | 0.004 | 2.00E-196 | 0.025 | 0.004 | 3.00E-12 |
| rs515135 | 2 | 2.1E+07 | T | C | 0.218 | 0.011 | 0.004 | 1.40E-02 | -0.140 | 0.005 | 1.00E-188 | -0.019 | 0.004 | 1.00E-05 |
| rs1260326 | 2 | 2.8E+07 | T | C | 0.413 | -0.011 | 0.003 | 1.00E-03 | 0.021 | 0.004 | 3.00E-08 | 0.110 | 0.003 | 2.00E-254 |
| rs3817588 | 2 | 2.8E+07 | T | C | 0.789 | -0.005 | 0.004 | 2.60E-01 | 0.026 | 0.005 | 3.00E-08 | 0.067 | 0.004 | 7.00E-58 |
| rs6544713 | 2 | 4.4E+07 | T | C | 0.294 | -0.003 | 0.004 | 4.30E-01 | 0.081 | 0.004 | 6.00E-85 | 0.013 | 0.004 | 7.00E-04 |
| rs4148218 | 2 | 4.4E+07 | A | G | 0.191 | 0.003 | 0.004 | 5.10E-01 | -0.044 | 0.005 | 3.00E-21 | -0.004 | 0.004 | 3.80E-01 |
| rs2710642 | 2 | 6.3E+07 | A | G | 0.619 | -0.010 | 0.004 | 6.80E-03 | 0.024 | 0.004 | 3.00E-10 | 0.007 | 0.003 | 6.00E-02 |
| rs17508045 | 2 | 1.2E+08 | T | C | 0.927 | -0.009 | 0.006 | 1.60E-01 | 0.049 | 0.007 | 9.00E-14 | -0.008 | 0.006 | 1.60E-01 |
| rs2030746 | 2 | 1.2E+08 | T | C | 0.398 | -0.003 | 0.004 | 4.90E-01 | 0.021 | 0.004 | 2.00E-08 | 0.003 | 0.004 | 3.80E-01 |
| rs16831243 | 2 | 1.4E+08 | T | C | 0.181 | 0.011 | 0.005 | 2.70E-02 | 0.038 | 0.006 | 8.00E-12 | -0.001 | 0.005 | 9.10E-01 |
| rs7607980 | 2 | 1.7E+08 | T | C | 0.851 | -0.045 | 0.005 | 1.00E-17 | 0.007 | 0.006 | 2.40E-01 | 0.036 | 0.005 | 7.00E-13 |
| rs355838 | 2 | 1.7E+08 | T | G | 0.376 | -0.019 | 0.003 | 4.00E-08 | 0.018 | 0.004 | 2.00E-06 | 0.014 | 0.004 | 8.00E-05 |
| rs2287623 | 2 | 1.7E+08 | A | G | 0.595 | -0.011 | 0.003 | 1.20E-03 | -0.022 | 0.004 | 7.00E-09 | 0.001 | 0.004 | 8.70E-01 |
| rs1047891 | 2 | 2.1E+08 | A | C | 0.302 | -0.027 | 0.004 | 5.00E-12 | 0.008 | 0.004 | 6.00E-02 | 0.000 | 100.000 | 1.00E+00 |
| rs1250229 | 2 | 2.2E+08 | T | C | 0.211 | 0.003 | 0.004 | 3.90E-01 | -0.024 | 0.004 | 8.00E-09 | -0.009 | 0.004 | 1.90E-02 |
| rs1515110 | 2 | 2.3E+08 | T | G | 0.619 | -0.032 | 0.003 | 2.00E-20 | 0.006 | 0.004 | 8.90E-02 | 0.027 | 0.003 | 5.00E-15 |
| rs11563251 | 2 | 2.3E+08 | T | C | 0.125 | 0.006 | 0.006 | 3.10E-01 | 0.035 | 0.006 | 2.00E-08 | 0.008 | 0.006 | 1.40E-01 |
| rs9875338 | 3 | 1.2E+07 | A | G | 0.388 | -0.007 | 0.003 | 3.40E-02 | -0.027 | 0.004 | 3.00E-13 | -0.014 | 0.003 | 2.00E-05 |
| rs7640978 | 3 | 3.3E+07 | T | C | 0.106 | 0.000 | 0.006 | 9.60E-01 | -0.039 | 0.007 | 1.00E-08 | -0.018 | 0.006 | 4.40E-03 |
| rs2290547 | 3 | 4.7E+07 | A | G | 0.211 | -0.030 | 0.005 | 8.00E-11 | 0.001 | 0.005 | 9.00E-01 | 0.010 | 0.004 | 3.00E-02 |
| rs2240327 | 3 | 5E+07 | A | G | 0.495 | -0.024 | 0.003 | 9.00E-13 | -0.001 | 0.003 | 8.80E-01 | 0.002 | 0.003 | 6.10E-01 |
| rs13326165 | 3 | 5.3E+07 | A | G | 0.187 | 0.029 | 0.004 | 2.00E-11 | -0.004 | 0.005 | 3.60E-01 | -0.021 | 0.004 | 9.00E-07 |
| rs6805251 | 3 | 1.2E+08 | T | C | 0.381 | 0.020 | 0.003 | 8.00E-09 | 0.012 | 0.004 | 1.30E-03 | -0.001 | 0.003 | 7.50E-01 |
| rs17345563 | 3 | 1.3E+08 | A | G | 0.871 | -0.014 | 0.005 | 7.00E-03 | 0.036 | 0.006 | 3.00E-10 | 0.015 | 0.005 | 3.80E-03 |
| rs687339 | 3 | 1.4E+08 | T | C | 0.767 | -0.032 | 0.004 | 3.00E-14 | 0.011 | 0.004 | 1.40E-02 | 0.029 | 0.004 | 6.00E-13 |
| rs1482852 | 3 | 1.6E+08 | A | G | 0.603 | -0.021 | 0.004 | 3.00E-09 | 0.003 | 0.004 | 4.50E-01 | 0.013 | 0.004 | 2.00E-04 |
| rs10513688 | 3 | 1.7E+08 | A | G | 0.110 | -0.005 | 0.006 | 3.80E-01 | 0.022 | 0.006 | 3.00E-04 | 0.031 | 0.006 | 4.00E-08 |
| rs6831256 | 4 | 3442937 | A | G | 0.591 | 0.013 | 0.004 | 3.00E-04 | -0.019 | 0.004 | 1.00E-06 | -0.026 | 0.003 | 9.00E-14 |
| rs10019888 | 4 | 2.6E+07 | A | G | 0.836 | 0.027 | 0.005 | 6.00E-09 | -0.018 | 0.005 | 3.00E-04 | -0.023 | 0.005 | 5.00E-07 |
| rs442177 | 4 | 8.8E+07 | T | G | 0.553 | -0.022 | 0.003 | 3.00E-10 | 0.016 | 0.004 | 2.00E-05 | 0.031 | 0.003 | 3.00E-20 |
| rs10029254 | 4 | 8.8E+07 | T | C | 0.216 | -0.009 | 0.004 | 3.70E-02 | 0.006 | 0.004 | 1.80E-01 | 0.027 | 0.004 | 1.00E-11 |
| rs3822072 | 4 | 9E+07 | A | G | 0.488 | -0.025 | 0.003 | 3.00E-13 | 0.007 | 0.004 | 4.60E-02 | 0.018 | 0.003 | 6.00E-08 |
| rs2602836 | 4 | 1E+08 | A | G | 0.427 | 0.019 | 0.003 | 2.00E-08 | -0.001 | 0.003 | 8.40E-01 | -0.009 | 0.003 | 7.00E-03 |
| rs13107325 | 4 | 1E+08 | T | C | 0.078 | -0.071 | 0.008 | 8.00E-20 | -0.016 | 0.009 | 6.10E-02 | 0.031 | 0.008 | 6.00E-05 |
| rs6450176 | 5 | 5.3E+07 | A | G | 0.278 | -0.025 | 0.004 | 1.00E-10 | 0.010 | 0.004 | 1.30E-02 | 0.019 | 0.004 | 6.00E-07 |
| rs9686661 | 5 | 5.6E+07 | T | C | 0.177 | -0.028 | 0.004 | 2.00E-10 | 0.018 | 0.005 | 2.00E-04 | 0.038 | 0.004 | 3.00E-18 |
| rs4976033 | 5 | 6.8E+07 | A | G | 0.620 | 0.022 | 0.004 | 9.00E-09 | 0.001 | 0.004 | 7.90E-01 | -0.014 | 0.004 | 1.00E-04 |
| rs7703051 | 5 | 7.5E+07 | A | C | 0.410 | 0.002 | 0.003 | 5.60E-01 | 0.073 | 0.004 | 5.00E-85 | 0.006 | 0.003 | 9.30E-02 |
| rs4530754 | 5 | 1.2E+08 | A | G | 0.582 | 0.001 | 0.003 | 8.10E-01 | 0.028 | 0.004 | 4.00E-14 | 0.002 | 0.003 | 6.40E-01 |
| rs6882076 | 5 | 1.6E+08 | T | C | 0.334 | -0.002 | 0.004 | 6.70E-01 | -0.046 | 0.004 | 5.00E-33 | -0.029 | 0.003 | 1.00E-16 |
| rs2294261 | 6 | 1.6E+07 | A | C | 0.487 | -0.009 | 0.003 | 1.50E-02 | 0.033 | 0.004 | 5.00E-19 | 0.002 | 0.003 | 5.40E-01 |
| rs1800562 | 6 | 2.6E+07 | A | G | 0.046 | -0.007 | 0.007 | 3.20E-01 | -0.062 | 0.008 | 2.00E-14 | 0.013 | 0.007 | 7.20E-02 |
| rs2247056 | 6 | 3.1E+07 | T | C | 0.218 | -0.012 | 0.004 | 2.30E-03 | -0.025 | 0.004 | 6.00E-09 | -0.038 | 0.004 | 2.00E-22 |
| rs205262 | 6 | 3.5E+07 | A | G | 0.734 | 0.028 | 0.004 | 2.00E-13 | 0.009 | 0.004 | 3.40E-02 | -0.003 | 0.004 | 4.50E-01 |
| rs998584 | 6 | 4.4E+07 | A | C | 0.515 | -0.026 | 0.004 | 4.00E-12 | 0.001 | 0.004 | 9.00E-01 | 0.029 | 0.004 | 2.00E-15 |
| rs17789218 | 6 | 1E+08 | T | C | 0.764 | -0.004 | 0.004 | 3.10E-01 | 0.024 | 0.004 | 3.00E-08 | 0.006 | 0.004 | 1.20E-01 |
| rs868943 | 6 | 1.2E+08 | A | G | 0.395 | -0.008 | 0.003 | 2.90E-02 | -0.026 | 0.004 | 1.00E-12 | -0.014 | 0.003 | 5.00E-05 |
| rs9491696 | 6 | 1.3E+08 | C | G | 0.548 | 0.020 | 0.003 | 3.00E-09 | -0.006 | 0.004 | 1.20E-01 | -0.018 | 0.003 | 9.00E-08 |
| rs634869 | 6 | 1.4E+08 | T | C | 0.438 | -0.023 | 0.003 | 8.00E-12 | 0.013 | 0.004 | 6.00E-04 | 0.027 | 0.003 | 4.00E-16 |
| rs12525163 | 6 | 1.5E+08 | T | C | 0.735 | -0.022 | 0.004 | 9.00E-09 | 0.004 | 0.004 | 2.90E-01 | 0.009 | 0.004 | 1.80E-02 |
| rs2297374 | 6 | 1.6E+08 | T | C | 0.385 | 0.006 | 0.004 | 1.10E-01 | -0.033 | 0.004 | 6.00E-18 | -0.009 | 0.003 | 7.70E-03 |
| rs1564348 | 6 | 1.6E+08 | T | C | 0.855 | 0.008 | 0.005 | 9.80E-02 | -0.048 | 0.005 | 3.00E-22 | -0.016 | 0.004 | 3.00E-04 |
| rs702485 | 7 | 6415797 | A | G | 0.550 | -0.024 | 0.003 | 1.00E-12 | -0.001 | 0.004 | 7.90E-01 | 0.002 | 0.003 | 6.40E-01 |
| rs17286602 | 7 | 1.6E+07 | A | T | 0.409 | 0.021 | 0.003 | 8.00E-10 | -0.003 | 0.004 | 3.80E-01 | -0.006 | 0.003 | 7.00E-02 |
| rs10282707 | 7 | 1.8E+07 | T | C | 0.397 | -0.025 | 0.003 | 8.00E-13 | -0.008 | 0.004 | 2.50E-02 | 0.009 | 0.003 | 7.20E-03 |
| rs12670798 | 7 | 2.2E+07 | T | C | 0.776 | 0.001 | 0.004 | 7.30E-01 | -0.034 | 0.004 | 7.00E-16 | -0.010 | 0.004 | 8.90E-03 |
| rs4722551 | 7 | 2.6E+07 | T | C | 0.830 | -0.010 | 0.005 | 2.70E-02 | -0.039 | 0.005 | 7.00E-16 | 0.027 | 0.005 | 2.00E-09 |
| rs2073547 | 7 | 4.5E+07 | A | G | 0.806 | 0.005 | 0.005 | 2.80E-01 | -0.049 | 0.005 | 5.00E-23 | -0.015 | 0.005 | 9.00E-04 |
| rs217386 | 7 | 4.5E+07 | A | G | 0.408 | 0.001 | 0.004 | 7.10E-01 | -0.036 | 0.004 | 8.00E-22 | -0.010 | 0.003 | 3.10E-03 |
| rs4917014 | 7 | 5E+07 | T | G | 0.660 | -0.022 | 0.004 | 8.00E-10 | -0.005 | 0.004 | 2.30E-01 | 0.001 | 0.004 | 7.40E-01 |
| rs17145738 | 7 | 7.3E+07 | T | C | 0.117 | 0.041 | 0.005 | 1.00E-14 | 0.004 | 0.006 | 5.00E-01 | -0.110 | 0.005 | 2.00E-103 |
| rs799160 | 7 | 7.3E+07 | T | C | 0.521 | -0.013 | 0.004 | 4.00E-04 | 0.005 | 0.004 | 2.50E-01 | 0.040 | 0.004 | 7.00E-29 |
| rs38855 | 7 | 1.2E+08 | A | G | 0.526 | -0.015 | 0.004 | 2.00E-05 | 0.001 | 0.004 | 7.80E-01 | 0.019 | 0.003 | 2.00E-08 |
| rs3996352 | 7 | 1.3E+08 | A | G | 0.542 | -0.030 | 0.003 | 4.00E-18 | 0.005 | 0.004 | 1.40E-01 | 0.018 | 0.003 | 7.00E-08 |
| rs17173637 | 7 | 1.5E+08 | T | C | 0.902 | 0.036 | 0.006 | 2.00E-10 | -0.007 | 0.006 | 2.60E-01 | -0.021 | 0.006 | 2.00E-04 |
| rs4240624 | 8 | 9221641 | A | G | 0.925 | 0.082 | 0.006 | 3.00E-45 | 0.067 | 0.006 | 7.00E-27 | -0.028 | 0.006 | 1.00E-06 |
| rs9693857 | 8 | 9304527 | T | C | 0.454 | -0.004 | 0.003 | 2.80E-01 | -0.005 | 0.004 | 2.10E-01 | 0.020 | 0.003 | 3.00E-09 |
| rs4332136 | 8 | 1.6E+07 | C | G | 0.017 | 0.480 | 0.065 | 1.00E-13 | -0.043 | 0.098 | 6.60E-01 | 0.024 | 0.053 | 6.50E-01 |
| rs4921914 | 8 | 1.8E+07 | T | C | 0.752 | -0.002 | 0.004 | 6.50E-01 | -0.023 | 0.004 | 2.00E-07 | -0.035 | 0.004 | 8.00E-19 |
| rs12678919 | 8 | 2E+07 | A | G | 0.879 | -0.160 | 0.006 | 5.00E-165 | 0.008 | 0.006 | 1.90E-01 | 0.170 | 0.006 | 2.00E-206 |
| rs894210 | 8 | 2E+07 | A | G | 0.565 | 0.069 | 0.003 | 1.00E-90 | -0.007 | 0.004 | 5.40E-02 | -0.067 | 0.003 | 5.00E-90 |
| rs10102164 | 8 | 5.6E+07 | A | G | 0.174 | -0.001 | 0.004 | 9.00E-01 | 0.032 | 0.005 | 3.00E-12 | 0.011 | 0.004 | 5.40E-03 |
| rs2326077 | 8 | 6E+07 | T | C | 0.648 | -0.004 | 0.004 | 2.20E-01 | -0.034 | 0.004 | 2.00E-19 | -0.018 | 0.003 | 2.00E-07 |
| rs2293889 | 8 | 1.2E+08 | T | G | 0.413 | -0.031 | 0.004 | 1.00E-18 | 0.015 | 0.004 | 1.10E-04 | 0.006 | 0.003 | 7.10E-02 |
| rs2737252 | 8 | 1.2E+08 | A | G | 0.256 | -0.013 | 0.004 | 9.00E-04 | -0.031 | 0.004 | 1.00E-14 | -0.009 | 0.004 | 1.30E-02 |
| rs4871137 | 8 | 1.2E+08 | T | G | 0.646 | -0.021 | 0.004 | 1.00E-08 | -0.004 | 0.004 | 2.80E-01 | -0.001 | 0.004 | 7.20E-01 |
| rs2980885 | 8 | 1.3E+08 | A | G | 0.782 | 0.035 | 0.004 | 4.00E-17 | -0.031 | 0.004 | 4.00E-12 | -0.058 | 0.004 | 5.00E-45 |
| rs2954022 | 8 | 1.3E+08 | A | C | 0.470 | 0.040 | 0.003 | 2.00E-32 | -0.055 | 0.004 | 4.00E-51 | -0.078 | 0.003 | 2.00E-124 |
| rs4075205 | 8 | 1.4E+08 | T | C | 0.549 | 0.022 | 0.003 | 2.00E-10 | -0.012 | 0.004 | 1.60E-03 | -0.009 | 0.003 | 8.30E-03 |
| rs7832643 | 8 | 1.5E+08 | T | G | 0.405 | -0.001 | 0.003 | 7.70E-01 | 0.034 | 0.004 | 7.00E-19 | 0.002 | 0.003 | 6.20E-01 |
| rs3780181 | 9 | 2630759 | A | G | 0.947 | 0.004 | 0.007 | 5.80E-01 | 0.045 | 0.007 | 1.00E-09 | -0.007 | 0.007 | 3.00E-01 |
| rs686030 | 9 | 1.5E+07 | A | C | 0.859 | 0.055 | 0.005 | 3.00E-29 | 0.009 | 0.005 | 1.10E-01 | 0.025 | 0.005 | 2.00E-07 |
| rs7033354 | 9 | 1.7E+07 | T | C | 0.646 | 0.015 | 0.003 | 1.00E-05 | -0.019 | 0.004 | 5.00E-07 | -0.019 | 0.003 | 3.00E-08 |
| rs1883025 | 9 | 1.1E+08 | T | C | 0.243 | -0.070 | 0.004 | 6.00E-66 | -0.030 | 0.004 | 1.00E-11 | -0.022 | 0.004 | 3.00E-08 |
| rs2472509 | 9 | 1.1E+08 | T | G | 0.639 | -0.023 | 0.004 | 7.00E-10 | 0.000 | 0.004 | 9.10E-01 | 0.002 | 0.004 | 5.10E-01 |
| rs8176720 | 9 | 1.4E+08 | T | C | 0.664 | 0.001 | 0.003 | 8.80E-01 | 0.033 | 0.004 | 6.00E-18 | -0.007 | 0.004 | 3.70E-02 |
| rs579459 | 9 | 1.4E+08 | T | C | 0.785 | -0.015 | 0.004 | 5.20E-04 | -0.067 | 0.005 | 3.00E-49 | 0.014 | 0.004 | 9.00E-04 |
| rs1781930 | 10 | 5186273 | A | G | 0.157 | -0.002 | 0.005 | 6.90E-01 | -0.010 | 0.005 | 3.50E-02 | -0.031 | 0.004 | 5.00E-13 |
| rs970548 | 10 | 4.5E+07 | A | C | 0.723 | -0.026 | 0.004 | 2.00E-11 | -0.016 | 0.004 | 2.00E-04 | -0.003 | 0.004 | 5.10E-01 |
| rs7897379 | 10 | 6.5E+07 | T | C | 0.532 | -0.019 | 0.003 | 1.00E-08 | -0.010 | 0.004 | 5.00E-03 | 0.027 | 0.003 | 2.00E-16 |
| rs2068888 | 10 | 9.5E+07 | A | G | 0.491 | 0.019 | 0.003 | 5.00E-08 | -0.017 | 0.004 | 1.00E-05 | -0.024 | 0.003 | 2.00E-12 |
| rs2255141 | 10 | 1.1E+08 | A | G | 0.319 | 0.034 | 0.004 | 1.00E-19 | 0.030 | 0.004 | 7.00E-14 | -0.021 | 0.004 | 1.00E-08 |
| rs2923084 | 11 | 1E+07 | A | G | 0.847 | 0.026 | 0.005 | 2.00E-08 | -0.012 | 0.005 | 1.30E-02 | -0.012 | 0.004 | 7.10E-03 |
| rs2303975 | 11 | 1.4E+07 | A | G | 0.143 | 0.028 | 0.005 | 1.00E-08 | -0.001 | 0.005 | 8.10E-01 | -0.012 | 0.005 | 1.60E-02 |
| rs10832962 | 11 | 1.9E+07 | T | C | 0.719 | 0.004 | 0.004 | 2.50E-01 | 0.032 | 0.004 | 2.00E-15 | 0.011 | 0.004 | 2.80E-03 |
| rs326214 | 11 | 4.7E+07 | A | G | 0.686 | -0.061 | 0.004 | 3.00E-42 | 0.007 | 0.005 | 1.40E-01 | 0.024 | 0.004 | 2.00E-08 |
| rs17788930 | 11 | 4.8E+07 | A | G | 0.643 | 0.036 | 0.004 | 1.00E-23 | 0.005 | 0.004 | 2.30E-01 | -0.011 | 0.003 | 1.30E-03 |
| rs11246602 | 11 | 5.1E+07 | T | C | 0.867 | -0.034 | 0.005 | 6.00E-11 | -0.002 | 0.006 | 7.30E-01 | 0.009 | 0.005 | 7.90E-02 |
| rs12226802 | 11 | 5.5E+07 | A | G | 0.868 | -0.033 | 0.005 | 2.00E-11 | 0.000 | 0.005 | 9.70E-01 | 0.007 | 0.005 | 1.80E-01 |
| rs174532 | 11 | 6.1E+07 | A | G | 0.293 | 0.021 | 0.004 | 8.00E-08 | 0.035 | 0.004 | 5.00E-17 | -0.016 | 0.004 | 3.00E-05 |
| rs1535 | 11 | 6.1E+07 | A | G | 0.637 | 0.039 | 0.004 | 5.00E-28 | 0.053 | 0.004 | 3.00E-43 | -0.046 | 0.003 | 1.00E-40 |
| rs12801636 | 11 | 6.5E+07 | A | G | 0.224 | 0.024 | 0.004 | 2.00E-08 | 0.008 | 0.004 | 7.90E-02 | -0.018 | 0.004 | 1.00E-05 |
| rs499974 | 11 | 7.5E+07 | A | C | 0.176 | -0.026 | 0.004 | 2.00E-09 | 0.001 | 0.005 | 7.90E-01 | -0.009 | 0.004 | 3.40E-02 |
| rs10790162 | 11 | 1.2E+08 | A | G | 0.091 | -0.095 | 0.007 | 3.00E-46 | 0.076 | 0.007 | 3.00E-26 | 0.230 | 0.006 | 1.00E-276 |
| rs603446 | 11 | 1.2E+08 | T | C | 0.447 | 0.002 | 0.003 | 6.00E-01 | -0.009 | 0.004 | 1.30E-02 | -0.050 | 0.003 | 2.00E-50 |
| rs7117842 | 11 | 1.2E+08 | T | C | 0.611 | -0.027 | 0.003 | 6.00E-15 | -0.019 | 0.004 | 3.00E-07 | 0.002 | 0.003 | 5.60E-01 |
| rs11220462 | 11 | 1.3E+08 | A | G | 0.143 | -0.016 | 0.006 | 4.00E-03 | 0.059 | 0.006 | 3.00E-23 | 0.019 | 0.005 | 4.00E-04 |
| rs11045163 | 12 | 2E+07 | A | G | 0.594 | -0.022 | 0.003 | 3.00E-10 | 0.006 | 0.004 | 1.40E-01 | 0.010 | 0.003 | 4.00E-03 |
| rs3741414 | 12 | 5.6E+07 | T | C | 0.191 | 0.030 | 0.004 | 2.00E-13 | -0.016 | 0.004 | 3.00E-04 | -0.028 | 0.004 | 8.00E-13 |
| rs10861661 | 12 | 1.1E+08 | A | C | 0.763 | 0.022 | 0.004 | 2.00E-07 | 0.000 | 0.005 | 9.30E-01 | -0.023 | 0.004 | 3.00E-08 |
| rs2241210 | 12 | 1.1E+08 | A | G | 0.447 | -0.033 | 0.003 | 3.00E-21 | -0.008 | 0.004 | 3.50E-02 | -0.003 | 0.003 | 3.80E-01 |
| rs653178 | 12 | 1.1E+08 | T | C | 0.532 | 0.026 | 0.003 | 1.00E-13 | 0.023 | 0.004 | 2.00E-09 | -0.010 | 0.003 | 4.00E-03 |
| rs6489818 | 12 | 1.1E+08 | A | G | 0.161 | 0.000 | 0.005 | 9.30E-01 | 0.028 | 0.005 | 6.00E-09 | -0.004 | 0.004 | 4.10E-01 |
| rs1186380 | 12 | 1.2E+08 | T | C | 0.234 | 0.000 | 0.004 | 9.60E-01 | -0.024 | 0.004 | 1.00E-08 | 0.003 | 0.004 | 5.00E-01 |
| rs1169288 | 12 | 1.2E+08 | A | C | 0.666 | -0.010 | 0.004 | 1.00E-02 | -0.038 | 0.004 | 9.00E-21 | -0.003 | 0.004 | 4.90E-01 |
| rs838876 | 12 | 1.2E+08 | A | G | 0.326 | 0.049 | 0.004 | 5.00E-36 | -0.003 | 0.004 | 4.70E-01 | -0.005 | 0.004 | 1.60E-01 |
| rs10773105 | 12 | 1.2E+08 | T | C | 0.517 | -0.036 | 0.003 | 1.00E-25 | 0.006 | 0.004 | 1.20E-01 | 0.004 | 0.003 | 2.80E-01 |
| rs4942486 | 13 | 3.2E+07 | T | C | 0.462 | -0.014 | 0.003 | 6.00E-05 | 0.024 | 0.004 | 3.00E-11 | 0.007 | 0.003 | 3.10E-02 |
| rs1341267 | 13 | 9.4E+07 | A | C | 0.578 | 0.002 | 0.003 | 5.00E-01 | 0.002 | 0.004 | 6.70E-01 | -0.018 | 0.003 | 4.00E-08 |
| rs8017377 | 14 | 2.4E+07 | A | G | 0.459 | -0.004 | 0.004 | 3.00E-01 | 0.030 | 0.004 | 3.00E-15 | 0.006 | 0.004 | 1.10E-01 |
| rs4983559 | 14 | 1E+08 | A | G | 0.623 | -0.020 | 0.004 | 4.00E-08 | -0.003 | 0.004 | 5.00E-01 | 0.000 | 0.004 | 9.80E-01 |
| rs2412710 | 15 | 4E+07 | A | G | 0.022 | -0.084 | 0.014 | 1.00E-09 | -0.002 | 0.015 | 8.70E-01 | 0.099 | 0.013 | 8.00E-14 |
| rs492571 | 15 | 4.2E+07 | T | C | 0.958 | 0.066 | 0.009 | 2.00E-13 | 0.003 | 0.010 | 7.30E-01 | -0.080 | 0.009 | 2.00E-19 |
| rs1532085 | 15 | 5.6E+07 | A | G | 0.367 | 0.110 | 0.004 | 2.00E-209 | 0.003 | 0.004 | 4.80E-01 | 0.031 | 0.003 | 5.00E-20 |
| rs261342 | 15 | 5.7E+07 | C | G | 0.793 | -0.110 | 0.006 | 6.00E-71 | 0.003 | 0.007 | 6.90E-01 | -0.045 | 0.006 | 4.00E-14 |
| rs2652834 | 15 | 6.1E+07 | A | G | 0.235 | -0.029 | 0.004 | 4.00E-11 | 0.002 | 0.005 | 6.80E-01 | 0.025 | 0.004 | 4.00E-09 |
| rs1035744 | 15 | 7E+07 | T | C | 0.745 | -0.006 | 0.004 | 1.50E-01 | 0.007 | 0.004 | 9.50E-02 | 0.021 | 0.004 | 4.00E-08 |
| rs3198697 | 16 | 1.5E+07 | T | C | 0.383 | 0.016 | 0.003 | 3.00E-06 | 0.010 | 0.004 | 1.00E-02 | -0.020 | 0.003 | 4.00E-09 |
| rs749671 | 16 | 3.1E+07 | A | G | 0.395 | 0.007 | 0.003 | 4.10E-02 | -0.015 | 0.004 | 4.00E-05 | -0.021 | 0.003 | 4.00E-10 |
| rs9930333 | 16 | 5.2E+07 | T | G | 0.552 | 0.020 | 0.004 | 1.00E-07 | 0.000 | 0.004 | 9.60E-01 | -0.021 | 0.004 | 1.00E-08 |
| rs9989419 | 16 | 5.6E+07 | A | G | 0.405 | -0.150 | 0.003 | 0.00E+00 | 0.028 | 0.004 | 8.00E-13 | 0.024 | 0.003 | 3.00E-12 |
| rs5880 | 16 | 5.6E+07 | C | G | 0.059 | -0.310 | 0.009 | 4.00E-257 | 0.047 | 0.010 | 9.00E-07 | 0.048 | 0.009 | 3.00E-08 |
| rs16942887 | 16 | 6.6E+07 | A | G | 0.133 | 0.083 | 0.005 | 1.00E-60 | 0.001 | 0.005 | 8.40E-01 | -0.012 | 0.005 | 2.00E-02 |
| rs2288002 | 16 | 7.1E+07 | A | G | 0.421 | -0.007 | 0.004 | 5.00E-02 | -0.029 | 0.004 | 5.00E-14 | -0.009 | 0.003 | 9.00E-03 |
| rs2000999 | 16 | 7.1E+07 | A | G | 0.185 | 0.002 | 0.004 | 5.90E-01 | 0.065 | 0.005 | 1.00E-45 | 0.019 | 0.004 | 9.00E-06 |
| rs2925979 | 16 | 8E+07 | T | C | 0.296 | -0.035 | 0.004 | 4.00E-21 | -0.003 | 0.004 | 4.40E-01 | 0.021 | 0.004 | 2.00E-08 |
| rs314253 | 17 | 7032374 | T | C | 0.665 | -0.003 | 0.004 | 4.00E-01 | 0.024 | 0.004 | 2.00E-10 | 0.009 | 0.003 | 1.20E-02 |
| rs4791641 | 17 | 8101874 | T | C | 0.544 | -0.004 | 0.003 | 2.30E-01 | -0.020 | 0.004 | 4.00E-08 | 0.003 | 0.003 | 4.00E-01 |
| rs931992 | 17 | 3.5E+07 | T | G | 0.675 | 0.034 | 0.004 | 3.00E-21 | 0.006 | 0.004 | 1.50E-01 | -0.008 | 0.004 | 1.80E-02 |
| rs8077889 | 17 | 3.9E+07 | A | C | 0.756 | 0.021 | 0.004 | 2.00E-06 | -0.001 | 0.004 | 9.10E-01 | -0.025 | 0.004 | 2.00E-09 |
| rs7225700 | 17 | 4.3E+07 | T | C | 0.327 | -0.010 | 0.004 | 6.00E-03 | -0.030 | 0.004 | 8.00E-15 | 0.005 | 0.004 | 1.90E-01 |
| rs4148005 | 17 | 6.4E+07 | T | G | 0.701 | 0.028 | 0.004 | 6.00E-15 | -0.015 | 0.003 | 1.00E-05 | -0.007 | 0.004 | 6.30E-02 |
| rs4969178 | 17 | 7.4E+07 | A | G | 0.373 | -0.026 | 0.003 | 4.00E-14 | -0.011 | 0.004 | 3.30E-03 | 0.018 | 0.004 | 3.00E-07 |
| rs4939883 | 18 | 4.5E+07 | T | C | 0.181 | -0.080 | 0.004 | 1.00E-71 | -0.021 | 0.005 | 1.00E-05 | -0.005 | 0.004 | 2.30E-01 |
| rs11660468 | 18 | 4.5E+07 | T | C | 0.397 | 0.039 | 0.003 | 9.00E-30 | 0.011 | 0.004 | 2.80E-03 | -0.001 | 0.004 | 8.20E-01 |
| rs952044 | 18 | 5.6E+07 | T | C | 0.342 | -0.023 | 0.004 | 3.00E-10 | -0.003 | 0.004 | 4.10E-01 | 0.010 | 0.004 | 4.30E-03 |
| rs2278236 | 19 | 8337581 | A | G | 0.544 | 0.033 | 0.004 | 7.00E-21 | 0.007 | 0.004 | 7.40E-02 | -0.014 | 0.003 | 4.00E-05 |
| rs6511720 | 19 | 1.1E+07 | T | G | 0.098 | 0.025 | 0.006 | 1.00E-05 | -0.220 | 0.006 | 3.00E-289 | -0.008 | 0.006 | 1.30E-01 |
| rs688 | 19 | 1.1E+07 | T | C | 0.447 | -0.011 | 0.004 | 2.00E-03 | 0.054 | 0.004 | 9.00E-48 | 0.004 | 0.003 | 2.20E-01 |
| rs10401969 | 19 | 1.9E+07 | T | C | 0.929 | -0.013 | 0.007 | 5.70E-02 | 0.120 | 0.007 | 2.00E-60 | 0.120 | 0.006 | 3.00E-76 |
| rs731839 | 19 | 3.9E+07 | A | G | 0.658 | 0.022 | 0.004 | 2.00E-09 | 0.002 | 0.004 | 6.60E-01 | -0.022 | 0.004 | 5.00E-10 |
| rs1688030 | 19 | 4E+07 | T | C | 0.057 | -0.009 | 0.007 | 2.20E-01 | -0.016 | 0.007 | 3.10E-02 | -0.038 | 0.007 | 3.00E-08 |
| rs6859 | 19 | 5E+07 | A | G | 0.442 | -0.018 | 0.004 | 1.00E-06 | 0.084 | 0.004 | 1.00E-101 | 0.014 | 0.003 | 6.00E-05 |
| rs7254892 | 19 | 5E+07 | A | G | 0.032 | 0.053 | 0.011 | 3.00E-06 | -0.490 | 0.010 | 0.00E+00 | 0.120 | 0.010 | 4.00E-31 |
| rs492602 | 19 | 5.4E+07 | A | G | 0.570 | 0.003 | 0.004 | 3.80E-01 | -0.029 | 0.004 | 3.00E-14 | -0.014 | 0.004 | 7.00E-05 |
| rs17695224 | 19 | 5.7E+07 | A | G | 0.239 | -0.029 | 0.004 | 2.00E-13 | -0.011 | 0.004 | 1.10E-02 | 0.012 | 0.004 | 2.10E-03 |
| rs103294 | 19 | 5.9E+07 | T | C | 0.186 | 0.052 | 0.004 | 4.00E-33 | 0.007 | 0.005 | 1.20E-01 | -0.002 | 0.004 | 6.10E-01 |
| rs364585 | 20 | 1.3E+07 | A | G | 0.367 | -0.001 | 0.003 | 8.80E-01 | -0.025 | 0.004 | 4.00E-11 | 0.002 | 0.003 | 6.00E-01 |
| rs2328223 | 20 | 1.8E+07 | A | C | 0.751 | 0.000 | 0.005 | 9.30E-01 | -0.030 | 0.005 | 2.00E-09 | 0.007 | 0.004 | 1.40E-01 |
| rs7264396 | 20 | 3.4E+07 | T | C | 0.219 | -0.005 | 0.004 | 1.90E-01 | -0.025 | 0.005 | 3.00E-08 | -0.011 | 0.004 | 8.00E-03 |
| rs6016381 | 20 | 3.9E+07 | T | C | 0.640 | -0.008 | 0.003 | 1.60E-02 | 0.036 | 0.004 | 6.00E-22 | 0.014 | 0.003 | 3.00E-05 |
| rs6065311 | 20 | 3.9E+07 | T | C | 0.540 | -0.002 | 0.003 | 4.80E-01 | -0.042 | 0.004 | 3.00E-30 | -0.006 | 0.003 | 6.70E-02 |
| rs1800961 | 20 | 4.2E+07 | T | C | 0.034 | -0.130 | 0.010 | 7.00E-38 | -0.069 | 0.011 | 1.00E-10 | -0.002 | 0.010 | 8.60E-01 |
| rs4465830 | 20 | 4.4E+07 | A | G | 0.798 | 0.060 | 0.004 | 4.00E-42 | -0.009 | 0.005 | 5.60E-02 | -0.053 | 0.004 | 5.00E-36 |
| rs181362 | 22 | 2E+07 | T | C | 0.194 | -0.038 | 0.004 | 7.00E-20 | -0.008 | 0.005 | 9.10E-02 | -0.010 | 0.004 | 2.00E-02 |
| rs5763662 | 22 | 2.9E+07 | T | C | 0.025 | 0.033 | 0.011 | 3.10E-03 | 0.077 | 0.012 | 2.00E-10 | 0.000 | 0.008 | 9.90E-01 |
| rs3761445 | 22 | 3.7E+07 | A | G | 0.615 | -0.016 | 0.004 | 5.00E-06 | 0.008 | 0.004 | 2.90E-02 | 0.023 | 0.003 | 7.00E-12 |

A total of 185 SNPs associated with serum high-density lipoprotein cholesterol (HDL-C), low-density lipoprotein cholesterol (LDL-C), and triglycerides concentrations were obtained from the GWAS summary results data published by the Global Lipids Genetics Consortium. These include 96 SNPs associated with HDL-C, 82 SNPs associated with LDL-C, and 60 SNPs associated with triglycerides. The associations with POAG were obtained from a GWAS using data from the UK Biobank.

POAG, primary open angle glaucoma; SNP, single nucleotide polymorphism; CHR, chromosome; BP, base pair; EA, effect allele; NEA, non effect allele; EAF, frequency of the effect allele from the corresponding study; β, the effect of the effect allele; se, the standard error of the beta; p, *P*-value from the GWAS.
