# Supplementary material for: Plasma lipid levels and risk of primary open angle glaucoma: a genetic study using Mendelian randomization
Source: BMC Ophthalmol. 2020 Oct 2;20:390. doi: 10.1186/s12886-020-01661-0 (PMC7532556; doi:10.1186/s12886-020-01661-0)
Supplement: Supplementary file 2 — Additional file 2: Table S2. Summary of selected instrumental variables for LDL-C. [file 12886_2020_1661_MOESM2_ESM.docx]

**Supplementary Table 2.** Summary of selected instrumental variables for LDL-C

| **SNP** | **EA_**  **exposure** | **NEA_**  **exposure** | **EA_**  **outcome** | **NEA_**  **outcome** | **β_**  **exposure** | **β_**  **outcome** | **EAF**  **_outcome** | **se_outcome** | **samplesize_outcome** | ***p*_**  **outcome** | **se_**  **exposure** | **samplesize_exposure** | ***p*_**  **exposure** |
| --- | --- | --- | --- | --- | --- | --- | --- | --- | --- | --- | --- | --- | --- |
| rs1010167 | C | G | C | G | -0.025 | 7.83E-07 | 0.612378 | 6.17E-05 | 463010 | 0.99 | 0.004 | 188577 | 2.05E-10 |
| rs10790162 | A | G | A | G | 0.076 | 8.31E-05 | 0.067884 | 0.000119 | 463010 | 0.49 | 0.007 | 188577 | 9.22E-28 |
| rs10832962 | T | C | T | C | 0.032 | -6.24E-05 | 0.74128 | 6.85E-05 | 463010 | 0.36 | 0.004 | 188577 | 6.22E-16 |
| rs10903129 | A | G | A | G | -0.033 | -3.35E-05 | 0.442955 | 6.02E-05 | 463010 | 0.58 | 0.004 | 188577 | 7.92E-17 |
| rs11563251 | T | C | T | C | 0.035 | 6.16E-05 | 0.111028 | 9.53E-05 | 463010 | 0.52 | 0.006 | 188577 | 2.72E-09 |
| rs1169288 | A | C | A | C | -0.038 | -4.99E-05 | 0.683656 | 6.48E-05 | 463010 | 0.44 | 0.004 | 188577 | 1.05E-21 |
| rs1250229 | T | C | T | C | -0.024 | -9.20E-05 | 0.261301 | 6.86E-05 | 463010 | 0.18 | 0.004 | 188577 | 9.87E-10 |
| rs1260326 | T | C | T | C | 0.021 | 5.50E-05 | 0.395707 | 6.12E-05 | 463010 | 0.37 | 0.004 | 188577 | 7.60E-08 |
| rs12670798 | T | C | T | C | -0.034 | -7.85E-05 | 0.753814 | 6.95E-05 | 463010 | 0.26 | 0.004 | 188577 | 9.48E-18 |
| rs1367117 | A | G | A | G | 0.12 | -6.05E-05 | 0.335116 | 6.34E-05 | 463010 | 0.34 | 0.004 | 188577 | 4.91E-198 |
| rs1535 | A | G | A | G | 0.053 | -2.34E-05 | 0.653513 | 6.30E-05 | 463010 | 0.71 | 0.004 | 188577 | 2.26E-40 |
| rs1564348 | T | C | T | C | -0.048 | 0.000126 | 0.830576 | 7.99E-05 | 463010 | 0.11 | 0.005 | 188577 | 4.00E-22 |
| rs16831243 | T | C | T | C | 0.038 | -2.63E-05 | 0.101615 | 9.83E-05 | 463010 | 0.79 | 0.006 | 188577 | 1.20E-10 |
| rs17345563 | A | G | A | G | 0.036 | -2.35E-05 | 0.88694 | 9.46E-05 | 463010 | 0.8 | 0.006 | 188577 | 9.87E-10 |
| rs17508045 | T | C | T | C | 0.049 | -6.81E-05 | 0.912531 | 0.000106 | 463010 | 0.52 | 0.007 | 188577 | 1.28E-12 |
| rs17789218 | T | C | T | C | 0.024 | 1.95E-05 | 0.755389 | 6.97E-05 | 463010 | 0.78 | 0.004 | 188577 | 9.87E-10 |
| rs1800562 | A | G | A | G | -0.062 | 0.000166 | 0.076777 | 0.000112 | 463010 | 0.14 | 0.008 | 188577 | 4.59E-15 |
| rs1800961 | T | C | T | C | -0.069 | 5.97E-07 | 0.030962 | 0.000173 | 463010 | 1 | 0.011 | 188577 | 1.77E-10 |
| rs1883025 | T | C | T | C | -0.03 | 5.14E-05 | 0.254668 | 6.87E-05 | 463010 | 0.45 | 0.004 | 188577 | 3.19E-14 |
| rs2000999 | A | G | A | G | 0.065 | -0.00018 | 0.189638 | 7.63E-05 | 463010 | 0.022 | 0.005 | 188577 | 6.12E-39 |
| rs2073547 | A | G | A | G | -0.049 | -4.60E-05 | 0.816041 | 7.68E-05 | 463010 | 0.55 | 0.005 | 188577 | 5.63E-23 |
| rs2255141 | A | G | A | G | 0.03 | 0.000112 | 0.276171 | 6.70E-05 | 463010 | 0.096 | 0.004 | 188577 | 3.19E-14 |
| rs2287623 | A | G | A | G | -0.022 | 2.08E-06 | 0.603098 | 6.13E-05 | 463010 | 0.97 | 0.004 | 188577 | 1.90E-08 |
| rs2294261 | A | C | A | C | 0.033 | -5.01E-05 | 0.509862 | 6.00E-05 | 463010 | 0.4 | 0.004 | 188577 | 7.92E-17 |
| rs2326077 | T | C | T | C | -0.034 | -7.12E-05 | 0.663339 | 6.34E-05 | 463010 | 0.26 | 0.004 | 188577 | 9.48E-18 |
| rs2328223 | A | C | A | C | -0.03 | -5.19E-05 | 0.812781 | 7.67E-05 | 463010 | 0.5 | 0.005 | 188577 | 9.87E-10 |
| rs2587534 | A | G | A | G | 0.039 | 6.01E-05 | 0.518744 | 6.02E-05 | 463010 | 0.32 | 0.004 | 188577 | 9.22E-23 |
| rs2642438 | A | G | A | G | -0.035 | 8.13E-05 | 0.297352 | 6.55E-05 | 463010 | 0.21 | 0.004 | 188577 | 1.07E-18 |
| rs267733 | A | G | A | G | 0.033 | -0.00016 | 0.839042 | 8.15E-05 | 463010 | 0.048 | 0.005 | 188577 | 2.06E-11 |
| rs2710642 | A | G | A | G | 0.024 | -1.36E-05 | 0.671714 | 6.37E-05 | 463010 | 0.83 | 0.004 | 188577 | 9.87E-10 |
| rs2737252 | A | G | A | G | -0.031 | -0.00015 | 0.280497 | 6.68E-05 | 463010 | 0.027 | 0.004 | 188577 | 4.59E-15 |
| rs2954022 | A | C | A | C | -0.055 | 3.98E-05 | 0.464625 | 6.01E-05 | 463010 | 0.51 | 0.004 | 188577 | 2.55E-43 |
| rs314253 | T | C | T | C | 0.024 | 6.67E-05 | 0.651017 | 6.28E-05 | 463010 | 0.29 | 0.004 | 188577 | 9.87E-10 |
| rs364585 | A | G | A | G | -0.025 | 1.13E-05 | 0.391261 | 6.14E-05 | 463010 | 0.85 | 0.004 | 188577 | 2.05E-10 |
| rs3780181 | A | G | A | G | 0.045 | -0.00024 | 0.932374 | 0.00012 | 463010 | 0.048 | 0.007 | 188577 | 6.44E-11 |
| rs4240624 | A | G | A | G | 0.067 | -3.56E-05 | 0.908834 | 0.000104 | 463010 | 0.73 | 0.006 | 188577 | 2.97E-29 |
| rs4530754 | A | G | A | G | 0.028 | -5.28E-05 | 0.544846 | 6.01E-05 | 463010 | 0.38 | 0.004 | 188577 | 1.28E-12 |
| rs4587594 | A | G | A | G | -0.049 | 8.80E-05 | 0.350988 | 6.28E-05 | 463010 | 0.16 | 0.004 | 188577 | 8.40E-35 |
| rs492602 | A | G | A | G | -0.029 | 6.98E-05 | 0.492224 | 5.98E-05 | 463010 | 0.24 | 0.004 | 188577 | 2.08E-13 |
| rs4942486 | T | C | T | C | 0.024 | -0.00013 | 0.476836 | 6.00E-05 | 463010 | 0.033 | 0.004 | 188577 | 9.87E-10 |
| rs5763662 | T | C | T | C | 0.077 | 3.73E-05 | 0.022519 | 0.000202 | 463010 | 0.85 | 0.012 | 188577 | 6.96E-11 |
| rs579459 | T | C | T | C | -0.067 | 3.72E-05 | 0.792668 | 7.39E-05 | 463010 | 0.61 | 0.005 | 188577 | 3.02E-41 |
| rs6065311 | T | C | T | C | -0.042 | 1.91E-05 | 0.521155 | 6.00E-05 | 463010 | 0.75 | 0.004 | 188577 | 4.32E-26 |
| rs6511720 | T | G | T | G | -0.22 | 8.75E-05 | 0.11881 | 9.26E-05 | 463010 | 0.35 | 0.006 | 188577 | 1.24E-294 |
| rs653178 | T | C | T | C | 0.023 | 1.46E-05 | 0.516524 | 5.99E-05 | 463010 | 0.81 | 0.004 | 188577 | 4.46E-09 |
| rs6544713 | T | C | T | C | 0.081 | 9.83E-05 | 0.323158 | 6.40E-05 | 463010 | 0.12 | 0.004 | 188577 | 1.78E-91 |
| rs6603981 | T | C | T | C | 0.034 | 8.79E-05 | 0.794812 | 7.39E-05 | 463010 | 0.23 | 0.004 | 188577 | 9.48E-18 |
| rs6882076 | T | C | T | C | -0.046 | -5.37E-05 | 0.365614 | 6.22E-05 | 463010 | 0.39 | 0.004 | 188577 | 6.60E-31 |
| rs7225700 | T | C | T | C | -0.03 | -3.68E-05 | 0.355386 | 6.27E-05 | 463010 | 0.56 | 0.004 | 188577 | 3.19E-14 |
| rs7254892 | A | G | A | G | -0.49 | 0.000198 | 0.03169 | 0.000171 | 463010 | 0.25 | 0.01 | 188577 | 0 |
| rs7640978 | T | C | T | C | -0.039 | -0.00014 | 0.08908 | 0.000105 | 463010 | 0.18 | 0.007 | 188577 | 1.26E-08 |
| rs7703051 | A | C | A | C | 0.073 | -1.39E-05 | 0.375395 | 6.18E-05 | 463010 | 0.82 | 0.004 | 188577 | 1.03E-74 |
| rs7832643 | T | G | T | G | 0.034 | -3.64E-05 | 0.403742 | 6.13E-05 | 463010 | 0.55 | 0.004 | 188577 | 9.48E-18 |
| rs8017377 | A | G | A | G | 0.03 | 5.13E-05 | 0.473973 | 6.00E-05 | 463010 | 0.39 | 0.004 | 188577 | 3.19E-14 |
| rs868943 | A | G | A | G | -0.026 | -8.88E-05 | 0.42376 | 6.06E-05 | 463010 | 0.14 | 0.004 | 188577 | 4.02E-11 |
| rs9875338 | A | G | A | G | -0.027 | -6.17E-05 | 0.400451 | 6.12E-05 | 463010 | 0.31 | 0.004 | 188577 | 7.39E-12 |
| rs9989419 | A | G | A | G | 0.028 | -0.00014 | 0.39396 | 6.13E-05 | 463010 | 0.025 | 0.004 | 188577 | 1.28E-12 |

LDL-C, low density lipoprotein cholesterol SNP, single nucleotide polymorphism; EA, effect allele; NEA, non effect allele; EAF, frequency of the effect allele from the corresponding study; β, the effect of the effect allele; se, the standard error of the beta; p, *P*-value from the GWAS.
