# Supplementary material for: Plasma lipid levels and risk of primary open angle glaucoma: a genetic study using Mendelian randomization
Source: BMC Ophthalmol. 2020 Oct 2;20:390. doi: 10.1186/s12886-020-01661-0 (PMC7532556; doi:10.1186/s12886-020-01661-0)
Supplement: Supplementary file 3 — Additional file 3: Table S3. Summary of selected instrumental variables for HDL-C. [file 12886_2020_1661_MOESM3_ESM.docx]

**Supplementary Table 3.** Summary of selected instrumental variables for HDL-C.

| **SNP** | **EA_**  **exposure** | **NEA_**  **exposure** | **EA_**  **outcome** | **NEA_**  **outcome** | **β_**  **exposure** | **β_**  **outcome** | **EAF**  **_outcome** | **se_outcome** | **samplesize_outcome** | ***p*_**  **outcome** | **se_**  **exposure** | **samplesize_exposure** | ***p*_**  **exposure** |
| --- | --- | --- | --- | --- | --- | --- | --- | --- | --- | --- | --- | --- | --- |
| rs10019888 | A | G | A | G | 0.027 | 3.03E-05 | 0.834511 | 8.10E-05 | 463010 | 0.71 | 0.005 | 188577 | 3.33E-08 |
| rs10282707 | T | C | T | C | -0.025 | 2.35E-05 | 0.397011 | 6.12E-05 | 463010 | 0.7 | 0.003 | 188577 | 3.93E-17 |
| rs103294 | T | C | T | C | 0.052 | -2.15E-05 | 0.223589 | 7.18E-05 | 463010 | 0.76 | 0.004 | 188577 | 6.12E-39 |
| rs1047891 | A | C | A | C | -0.027 | 3.40E-05 | 0.315766 | 6.44E-05 | 463010 | 0.6 | 0.004 | 188577 | 7.39E-12 |
| rs10790162 | A | G | A | G | -0.095 | 8.31E-05 | 0.067884 | 0.000119 | 463010 | 0.49 | 0.007 | 188577 | 2.96E-42 |
| rs11045163 | A | G | A | G | -0.022 | -4.94E-05 | 0.569393 | 6.06E-05 | 463010 | 0.42 | 0.003 | 188577 | 1.12E-13 |
| rs12133576 | A | G | A | G | 0.024 | 3.20E-05 | 0.368943 | 6.20E-05 | 463010 | 0.61 | 0.003 | 188577 | 6.22E-16 |
| rs12145743 | T | G | T | G | -0.02 | 5.28E-05 | 0.662605 | 6.33E-05 | 463010 | 0.4 | 0.004 | 188577 | 2.87E-07 |
| rs12525163 | T | C | T | C | -0.022 | -0.00012 | 0.726639 | 6.73E-05 | 463010 | 0.071 | 0.004 | 188577 | 1.90E-08 |
| rs12678919 | A | G | A | G | -0.16 | -4.86E-05 | 0.902613 | 0.000101 | 463010 | 0.63 | 0.006 | 188577 | 5.73E-157 |
| rs13107325 | T | C | T | C | -0.071 | 9.26E-05 | 0.07491 | 0.000114 | 463010 | 0.42 | 0.008 | 188577 | 3.50E-19 |
| rs1367117 | A | G | A | G | -0.022 | -6.05E-05 | 0.335116 | 6.34E-05 | 463010 | 0.34 | 0.004 | 188577 | 1.90E-08 |
| rs1482852 | A | G | A | G | -0.021 | 7.28E-05 | 0.597959 | 6.13E-05 | 463010 | 0.23 | 0.004 | 188577 | 7.60E-08 |
| rs1515110 | T | G | T | G | -0.032 | -8.39E-05 | 0.636731 | 6.23E-05 | 463010 | 0.18 | 0.003 | 188577 | 7.29E-27 |
| rs1532085 | A | G | A | G | 0.11 | 8.73E-05 | 0.386464 | 6.16E-05 | 463010 | 0.16 | 0.004 | 188577 | 8.78E-167 |
| rs1535 | A | G | A | G | 0.039 | -2.34E-05 | 0.653513 | 6.30E-05 | 463010 | 0.71 | 0.004 | 188577 | 9.22E-23 |
| rs1689797 | A | C | A | C | -0.036 | 2.46E-05 | 0.333474 | 6.36E-05 | 463010 | 0.7 | 0.004 | 188577 | 1.13E-19 |
| rs16942887 | A | G | A | G | 0.083 | -6.95E-05 | 0.115118 | 9.38E-05 | 463010 | 0.46 | 0.005 | 188577 | 3.48E-62 |
| rs17145738 | T | C | T | C | 0.041 | 0.000117 | 0.122279 | 9.13E-05 | 463010 | 0.2 | 0.005 | 188577 | 1.20E-16 |
| rs17173637 | T | C | T | C | 0.036 | 9.72E-05 | 0.915606 | 0.000108 | 463010 | 0.37 | 0.006 | 188577 | 9.87E-10 |
| rs17695224 | A | G | A | G | -0.029 | -7.92E-05 | 0.266863 | 6.78E-05 | 463010 | 0.24 | 0.004 | 188577 | 2.08E-13 |
| rs181362 | T | C | T | C | -0.038 | 4.38E-05 | 0.19235 | 7.59E-05 | 463010 | 0.56 | 0.004 | 188577 | 1.05E-21 |
| rs1883025 | T | C | T | C | -0.07 | 5.14E-05 | 0.254668 | 6.87E-05 | 463010 | 0.45 | 0.004 | 188577 | 7.16E-69 |
| rs205262 | A | G | A | G | 0.028 | 6.81E-05 | 0.730992 | 6.76E-05 | 463010 | 0.31 | 0.004 | 188577 | 1.28E-12 |
| rs2068888 | A | G | A | G | 0.019 | 3.67E-05 | 0.450556 | 6.02E-05 | 463010 | 0.54 | 0.003 | 188577 | 1.20E-10 |
| rs2240327 | A | G | A | G | -0.024 | -4.79E-05 | 0.522659 | 5.99E-05 | 463010 | 0.42 | 0.003 | 188577 | 6.22E-16 |
| rs2241210 | A | G | A | G | -0.033 | 8.76E-05 | 0.475988 | 6.00E-05 | 463010 | 0.14 | 0.003 | 188577 | 1.91E-28 |
| rs2255141 | A | G | A | G | 0.034 | 0.000112 | 0.276171 | 6.70E-05 | 463010 | 0.096 | 0.004 | 188577 | 9.48E-18 |
| rs2278236 | A | G | A | G | 0.033 | -0.00013 | 0.516539 | 6.00E-05 | 463010 | 0.032 | 0.004 | 188577 | 7.92E-17 |
| rs2290547 | A | G | A | G | -0.03 | 1.19E-05 | 0.174179 | 7.89E-05 | 463010 | 0.88 | 0.005 | 188577 | 9.87E-10 |
| rs2293889 | T | G | T | G | -0.031 | 6.11E-05 | 0.432419 | 6.06E-05 | 463010 | 0.31 | 0.004 | 188577 | 4.59E-15 |
| rs2303975 | A | G | A | G | 0.028 | -7.42E-05 | 0.111247 | 9.55E-05 | 463010 | 0.44 | 0.005 | 188577 | 1.07E-08 |
| rs2602836 | A | G | A | G | 0.019 | -2.57E-05 | 0.420402 | 6.08E-05 | 463010 | 0.67 | 0.003 | 188577 | 1.20E-10 |
| rs2642438 | A | G | A | G | -0.03 | 8.13E-05 | 0.297352 | 6.55E-05 | 463010 | 0.21 | 0.004 | 188577 | 3.19E-14 |
| rs2923084 | A | G | A | G | 0.026 | -1.08E-05 | 0.821333 | 7.82E-05 | 463010 | 0.89 | 0.005 | 188577 | 9.96E-08 |
| rs2925979 | T | C | T | C | -0.035 | -4.73E-05 | 0.299962 | 6.54E-05 | 463010 | 0.47 | 0.004 | 188577 | 1.07E-18 |
| rs2954022 | A | C | A | C | 0.04 | 3.98E-05 | 0.464625 | 6.01E-05 | 463010 | 0.51 | 0.003 | 188577 | 7.41E-41 |
| rs326214 | A | G | A | G | -0.061 | -7.49E-05 | 0.67743 | 6.40E-05 | 463010 | 0.24 | 0.004 | 188577 | 8.23E-53 |
| rs3741414 | T | C | T | C | 0.03 | -9.69E-06 | 0.240638 | 7.00E-05 | 463010 | 0.89 | 0.004 | 188577 | 3.19E-14 |
| rs3822072 | A | G | A | G | -0.025 | -8.25E-05 | 0.45108 | 6.02E-05 | 463010 | 0.17 | 0.003 | 188577 | 3.93E-17 |
| rs3996352 | A | G | A | G | -0.03 | -2.17E-06 | 0.508216 | 6.00E-05 | 463010 | 0.97 | 0.003 | 188577 | 7.62E-24 |
| rs4075205 | T | C | T | C | 0.022 | -5.46E-05 | 0.537151 | 6.01E-05 | 463010 | 0.36 | 0.003 | 188577 | 1.12E-13 |
| rs4148005 | T | G | T | G | 0.028 | -3.34E-05 | 0.682579 | 6.44E-05 | 463010 | 0.6 | 0.004 | 188577 | 1.28E-12 |
| rs4240624 | A | G | A | G | 0.082 | -3.56E-05 | 0.908834 | 0.000104 | 463010 | 0.73 | 0.006 | 188577 | 8.03E-43 |
| rs4465830 | A | G | A | G | 0.06 | -8.68E-05 | 0.813483 | 7.69E-05 | 463010 | 0.26 | 0.004 | 188577 | 3.67E-51 |
| rs4650994 | A | G | A | G | -0.021 | -7.16E-05 | 0.531646 | 5.99E-05 | 463010 | 0.23 | 0.003 | 188577 | 1.28E-12 |
| rs4660293 | A | G | A | G | 0.035 | 1.44E-05 | 0.765753 | 7.08E-05 | 463010 | 0.84 | 0.004 | 188577 | 1.07E-18 |
| rs4846914 | A | G | A | G | 0.048 | 1.11E-05 | 0.605878 | 6.14E-05 | 463010 | 0.86 | 0.003 | 188577 | 6.39E-58 |
| rs4917014 | T | G | T | G | -0.022 | 1.89E-05 | 0.68115 | 6.44E-05 | 463010 | 0.77 | 0.004 | 188577 | 1.90E-08 |
| rs492571 | T | C | T | C | 0.066 | -9.51E-05 | 0.9576 | 0.000149 | 463010 | 0.52 | 0.009 | 188577 | 1.12E-13 |
| rs4939883 | T | C | T | C | -0.08 | 5.76E-05 | 0.179505 | 7.81E-05 | 463010 | 0.46 | 0.004 | 188577 | 2.75E-89 |
| rs4969178 | A | G | A | G | -0.026 | -8.72E-05 | 0.389289 | 6.22E-05 | 463010 | 0.16 | 0.003 | 188577 | 2.22E-18 |
| rs4976033 | A | G | A | G | 0.022 | -3.72E-05 | 0.598042 | 6.19E-05 | 463010 | 0.55 | 0.004 | 188577 | 1.90E-08 |
| rs4983559 | A | G | A | G | -0.02 | -5.83E-05 | 0.613578 | 6.16E-05 | 463010 | 0.34 | 0.004 | 188577 | 2.87E-07 |
| rs499974 | A | C | A | C | -0.026 | -0.00011 | 0.157052 | 8.23E-05 | 463010 | 0.17 | 0.004 | 188577 | 4.02E-11 |
| rs634869 | T | C | T | C | -0.023 | 3.65E-05 | 0.409163 | 6.09E-05 | 463010 | 0.55 | 0.003 | 188577 | 8.83E-15 |
| rs646776 | T | C | T | C | -0.034 | -0.00019 | 0.777917 | 7.20E-05 | 463010 | 0.0096 | 0.004 | 188577 | 9.48E-18 |
| rs6805251 | T | C | T | C | 0.02 | -0.00015 | 0.382962 | 6.16E-05 | 463010 | 0.016 | 0.003 | 188577 | 1.31E-11 |
| rs686030 | A | C | A | C | 0.055 | -5.38E-05 | 0.858633 | 8.61E-05 | 463010 | 0.53 | 0.005 | 188577 | 1.91E-28 |
| rs687339 | T | C | T | C | -0.032 | -2.84E-05 | 0.771609 | 7.14E-05 | 463010 | 0.69 | 0.004 | 188577 | 6.22E-16 |
| rs702485 | A | G | A | G | -0.024 | 2.64E-05 | 0.545384 | 6.01E-05 | 463010 | 0.66 | 0.003 | 188577 | 6.22E-16 |
| rs7117842 | T | C | T | C | -0.027 | -3.62E-05 | 0.630963 | 6.24E-05 | 463010 | 0.56 | 0.003 | 188577 | 1.13E-19 |
| rs731839 | A | G | A | G | 0.022 | 4.74E-05 | 0.665376 | 6.35E-05 | 463010 | 0.46 | 0.004 | 188577 | 1.90E-08 |
| rs7607980 | T | C | T | C | -0.045 | -8.45E-05 | 0.879619 | 9.22E-05 | 463010 | 0.36 | 0.005 | 188577 | 1.13E-19 |
| rs7897379 | T | C | T | C | -0.019 | -0.00013 | 0.521583 | 6.00E-05 | 463010 | 0.034 | 0.003 | 188577 | 1.20E-10 |
| rs838876 | A | G | A | G | 0.049 | -8.22E-05 | 0.318512 | 6.50E-05 | 463010 | 0.21 | 0.004 | 188577 | 8.40E-35 |
| rs931992 | T | G | T | G | 0.034 | -7.45E-05 | 0.667025 | 6.36E-05 | 463010 | 0.24 | 0.004 | 188577 | 9.48E-18 |
| rs9491696 | C | G | C | G | 0.02 | -0.00012 | 0.50737 | 6.00E-05 | 463010 | 0.041 | 0.003 | 188577 | 1.31E-11 |
| rs952044 | T | C | T | C | -0.023 | 0.000129 | 0.329165 | 6.41E-05 | 463010 | 0.045 | 0.004 | 188577 | 4.46E-09 |
| rs9686661 | T | C | T | C | -0.028 | 7.02E-05 | 0.200616 | 7.47E-05 | 463010 | 0.35 | 0.004 | 188577 | 1.28E-12 |
| rs970548 | A | C | A | C | -0.026 | -2.85E-05 | 0.753378 | 6.95E-05 | 463010 | 0.68 | 0.004 | 188577 | 4.02E-11 |
| rs998584 | A | C | A | C | -0.026 | -5.31E-05 | 0.482776 | 6.01E-05 | 463010 | 0.38 | 0.004 | 188577 | 4.02E-11 |
| rs9989419 | A | G | A | G | -0.15 | -0.00014 | 0.39396 | 6.13E-05 | 463010 | 0.025 | 0.003 | 188577 | 3.00E-12 |

HDL-C, high density lipoprotein cholesterol; SNP, single nucleotide polymorphism; EA, effect allele; NEA, non effect allele; EAF, frequency of the effect allele from the corresponding study; β, the effect of the effect allele; se, the standard error of the beta; p, *P*-value from the GWAS.
