# Supplementary material for: Plasma lipid levels and risk of primary open angle glaucoma: a genetic study using Mendelian randomization
Source: BMC Ophthalmol. 2020 Oct 2;20:390. doi: 10.1186/s12886-020-01661-0 (PMC7532556; doi:10.1186/s12886-020-01661-0)
Supplement: Supplementary file 4 — Additional file 4: Table S4. Summary of selected instrumental variables for TG. [file 12886_2020_1661_MOESM4_ESM.docx]

**Supplementary Table 4.** Summary of selected instrumental variables for TG.

| **SNP** | **EA_**  **exposure** | **NEA_**  **exposure** | **EA_**  **outcome** | **NEA_**  **outcome** | **β_**  **exposure** | **β_**  **outcome** | **EAF**  **_outcome** | **se_outcome** | **Sample size_outcome** | ***p*_**  **outcome** | **se_**  **exposure** | **samplesize_exposure** | ***p*_**  **exposure** |
| --- | --- | --- | --- | --- | --- | --- | --- | --- | --- | --- | --- | --- | --- |
| rs1035744 | T | C | T | C | 0.021 | -1.64E-05 | 0.730288 | 6.78E-05 | 463010 | 0.81 | 0.004 | 188577 | 7.60E-08 |
| rs10401969 | T | C | T | C | 0.12 | -1.52E-05 | 0.923826 | 0.000113 | 463010 | 0.89 | 0.006 | 188577 | 2.75E-89 |
| rs10513688 | A | G | A | G | 0.031 | -4.69E-05 | 0.097324 | 0.000101 | 463010 | 0.64 | 0.006 | 188577 | 1.19E-07 |
| rs10790162 | A | G | A | G | 0.23 | 8.31E-05 | 0.067884 | 0.000119 | 463010 | 0.49 | 0.006 | 188577 | 1.00E-276 |
| rs10861661 | A | C | A | C | -0.023 | 4.96E-06 | 0.753994 | 6.96E-05 | 463010 | 0.94 | 0.004 | 188577 | 4.46E-09 |
| rs1260326 | T | C | T | C | 0.11 | 5.50E-05 | 0.395707 | 6.12E-05 | 463010 | 0.37 | 0.003 | 188577 | 1.24E-294 |
| rs12678919 | A | G | A | G | 0.17 | -4.86E-05 | 0.902613 | 0.000101 | 463010 | 0.63 | 0.006 | 188577 | 6.72E-177 |
| rs1341267 | A | C | A | C | -0.018 | -3.75E-05 | 0.599026 | 6.11E-05 | 463010 | 0.54 | 0.003 | 188577 | 9.87E-10 |
| rs1367117 | A | G | A | G | 0.025 | -6.05E-05 | 0.335116 | 6.34E-05 | 463010 | 0.34 | 0.004 | 188577 | 2.05E-10 |
| rs1515110 | T | G | T | G | 0.027 | -8.39E-05 | 0.636731 | 6.23E-05 | 463010 | 0.18 | 0.003 | 188577 | 1.13E-19 |
| rs1532085 | A | G | A | G | 0.031 | 8.73E-05 | 0.386464 | 6.16E-05 | 463010 | 0.16 | 0.003 | 188577 | 2.49E-25 |
| rs1535 | A | G | A | G | -0.046 | -2.34E-05 | 0.653513 | 6.30E-05 | 463010 | 0.71 | 0.003 | 188577 | 2.29E-53 |
| rs17145738 | T | C | T | C | -0.11 | 0.000117 | 0.122279 | 9.13E-05 | 463010 | 0.2 | 0.005 | 188577 | 1.44E-107 |
| rs1781930 | A | G | A | G | -0.031 | 6.77E-05 | 0.177771 | 7.84E-05 | 463010 | 0.39 | 0.004 | 188577 | 4.59E-15 |
| rs1883025 | T | C | T | C | -0.022 | 5.14E-05 | 0.254668 | 6.87E-05 | 463010 | 0.45 | 0.004 | 188577 | 1.90E-08 |
| rs2068888 | A | G | A | G | -0.024 | 3.67E-05 | 0.450556 | 6.02E-05 | 463010 | 0.54 | 0.003 | 188577 | 6.22E-16 |
| rs2247056 | T | C | T | C | -0.038 | -4.32E-05 | 0.296745 | 6.55E-05 | 463010 | 0.51 | 0.004 | 188577 | 1.05E-21 |
| rs2255141 | A | G | A | G | -0.021 | 0.000112 | 0.276171 | 6.70E-05 | 463010 | 0.096 | 0.004 | 188577 | 7.60E-08 |
| rs2925979 | T | C | T | C | 0.021 | -4.73E-05 | 0.299962 | 6.54E-05 | 463010 | 0.47 | 0.004 | 188577 | 7.60E-08 |
| rs2954022 | A | C | A | C | -0.078 | 3.98E-05 | 0.464625 | 6.01E-05 | 463010 | 0.51 | 0.003 | 188577 | 2.48E-149 |
| rs3198697 | T | C | T | C | -0.02 | 9.92E-05 | 0.407573 | 6.09E-05 | 463010 | 0.1 | 0.003 | 188577 | 1.31E-11 |
| rs326214 | A | G | A | G | 0.024 | -7.49E-05 | 0.67743 | 6.40E-05 | 463010 | 0.24 | 0.004 | 188577 | 9.87E-10 |
| rs3741414 | T | C | T | C | -0.028 | -9.69E-06 | 0.240638 | 7.00E-05 | 463010 | 0.89 | 0.004 | 188577 | 1.28E-12 |
| rs3761445 | A | G | A | G | 0.023 | -3.72E-05 | 0.5988 | 6.12E-05 | 463010 | 0.54 | 0.003 | 188577 | 8.83E-15 |
| rs38855 | A | G | A | G | 0.019 | -0.00016 | 0.529227 | 6.03E-05 | 463010 | 0.0068 | 0.003 | 188577 | 1.20E-10 |
| rs442177 | T | G | T | G | 0.031 | -4.65E-05 | 0.594454 | 6.10E-05 | 463010 | 0.45 | 0.003 | 188577 | 2.49E-25 |
| rs4465830 | A | G | A | G | -0.053 | -8.68E-05 | 0.813483 | 7.69E-05 | 463010 | 0.26 | 0.004 | 188577 | 2.26E-40 |
| rs4587594 | A | G | A | G | -0.069 | 8.80E-05 | 0.350988 | 6.28E-05 | 463010 | 0.16 | 0.003 | 188577 | 2.33E-117 |
| rs4722551 | T | C | T | C | 0.027 | -1.39E-05 | 0.841355 | 8.19E-05 | 463010 | 0.87 | 0.005 | 188577 | 3.33E-08 |
| rs4846914 | A | G | A | G | -0.04 | 1.11E-05 | 0.605878 | 6.14E-05 | 463010 | 0.86 | 0.003 | 188577 | 7.41E-41 |
| rs492571 | T | C | T | C | -0.08 | -9.51E-05 | 0.9576 | 0.000149 | 463010 | 0.52 | 0.009 | 188577 | 3.09E-19 |
| rs634869 | T | C | T | C | 0.027 | 3.65E-05 | 0.409163 | 6.09E-05 | 463010 | 0.55 | 0.003 | 188577 | 1.13E-19 |
| rs6831256 | A | G | A | G | -0.026 | 0.000114 | 0.576759 | 6.07E-05 | 463010 | 0.061 | 0.003 | 188577 | 2.22E-18 |
| rs687339 | T | C | T | C | 0.029 | -2.84E-05 | 0.771609 | 7.14E-05 | 463010 | 0.69 | 0.004 | 188577 | 2.08E-13 |
| rs6882076 | T | C | T | C | -0.029 | -5.37E-05 | 0.365614 | 6.22E-05 | 463010 | 0.39 | 0.003 | 188577 | 2.09E-22 |
| rs7033354 | T | C | T | C | -0.019 | -4.87E-05 | 0.678428 | 6.41E-05 | 463010 | 0.45 | 0.003 | 188577 | 1.20E-10 |
| rs7254892 | A | G | A | G | 0.12 | 0.000198 | 0.03169 | 0.000171 | 463010 | 0.25 | 0.01 | 188577 | 1.78E-33 |
| rs731839 | A | G | A | G | -0.022 | 4.74E-05 | 0.665376 | 6.35E-05 | 463010 | 0.46 | 0.004 | 188577 | 1.90E-08 |
| rs749671 | A | G | A | G | -0.021 | -5.39E-05 | 0.372287 | 6.19E-05 | 463010 | 0.38 | 0.003 | 188577 | 1.28E-12 |
| rs7607980 | T | C | T | C | 0.036 | -8.45E-05 | 0.879619 | 9.22E-05 | 463010 | 0.36 | 0.005 | 188577 | 3.01E-13 |
| rs7897379 | T | C | T | C | 0.027 | -0.00013 | 0.521583 | 6.00E-05 | 463010 | 0.034 | 0.003 | 188577 | 1.13E-19 |
| rs8077889 | A | C | A | C | -0.025 | 2.08E-05 | 0.78542 | 7.30E-05 | 463010 | 0.78 | 0.004 | 188577 | 2.05E-10 |
| rs9686661 | T | C | T | C | 0.038 | 7.02E-05 | 0.200616 | 7.47E-05 | 463010 | 0.35 | 0.004 | 188577 | 1.05E-21 |
| rs9693857 | T | C | T | C | 0.02 | -4.98E-05 | 0.445871 | 6.05E-05 | 463010 | 0.41 | 0.003 | 188577 | 1.31E-11 |
| rs998584 | A | C | A | C | 0.029 | -5.31E-05 | 0.482776 | 6.01E-05 | 463010 | 0.38 | 0.004 | 188577 | 2.08E-13 |
| rs9989419 | A | G | A | G | 0.024 | -0.00014 | 0.39396 | 6.13E-05 | 463010 | 0.025 | 0.003 | 188577 | 6.22E-16 |

TG, triglycerides; SNP, single nucleotide polymorphism; EA, effect allele; NEA, non effect allele; EAF, frequency of the effect allele from the corresponding study; β, the effect of the effect allele; se, the standard error of the beta; p, *P*-value from the GWAS.
